# An Overview of Antimicrobial Resistance Profiles of Publicly Available *Salmonella* Genomes with Sufficient Quality and Metadata

**DOI:** 10.1089/fpd.2022.0080

**Published:** 2023-09-04

**Authors:** Narong Nuanmuang, Pimlapas Leekitcharoenphon, Patrick Murigu Kamau Njage, Alexander Gmeiner, Frank M. Aarestrup

**Affiliations:** Research Group for Genomic Epidemiology, National Food Institute, Technical University of Denmark, Kgs. Lyngby, Denmark.

**Keywords:** *Salmonella enterica*, whole genome sequencing, antimicrobial resistance, antibiotic resistance genes, WGS, AMR

## Abstract

(*S*. *enterica*) is a commensal organism or pathogen causing diseases in animals and humans, as well as widespread in the environment. Antimicrobial resistance (AMR) has increasingly affected both animal and human health and continues to raise public health concerns. A decade ago, it was estimated that the increased use of whole genome sequencing (WGS) combined with sharing of public data would drastically change and improve the surveillance and understanding of *Salmonella* epidemiology and AMR. This study aimed to evaluate the current usefulness of public WGS data for *Salmonella* surveillance and to investigate the associations between serovars, antibiotic resistance genes (ARGs), and metadata. Out of 191,306 *Salmonella* genomes deposited in European Nucleotide Archive and NCBI databases, 47,452 WGS with sufficient minimum metadata (country, year, and source) of *S. enterica* were retrieved from 116 countries and isolated between 1905 and 2020. For *in silico* analysis of the WGS data, KmerFinder, SISTR, and ResFinder were used for species, serovars, and AMR identification, respectively. The results showed that the five common isolation sources of *S. enterica* are human (29.10%), avian (22.50%), environment (11.89%), water (9.33%), and swine (6.62%). The most common ARG profiles for each class of antimicrobials are β-lactam (*bla*_TEM-1B_; 6.78%), fluoroquinolone [(*parC*[T57S], *qnrB19*); 0.87%], folate pathway antagonist (*sul2*; 8.35%), macrolide [*mph*(A); 0.39%], phenicol (*floR*; 5.94%), polymyxin B (*mcr-1.1*; 0.09%), and tetracycline [*tet*(A); 12.95%]. Our study reports the first overview of ARG profiles in publicly available *Salmonella* genomes from online databases. All data sets from this study can be searched at Microreact.

## Introduction

*S**almonella enterica* can be found as a commensal organism in a wide range of hosts, especially in food animals, and is also a leading pathogen in animals and humans (Eng et al., 2015; Ferrari et al., 2019). It causes foodborne illnesses ranging from mild diarrhea and gastroenteritis to severe systemic infections (Eng et al., 2015). Antimicrobial resistance (AMR) in *Salmonella* has continuously been reported and continues to raise a public health concern (CDC, 2019; FDA, 2022; Lauteri et al., 2022).

More than 2600 *S. enterica* serovars have been identified, *S. enterica* serovars Enteritidis and Typhimurium are the most commonly reported serovars causing human salmonellosis; however, other serovars appear to be more prevalent in other regions (Hendriksen et al., 2011). Changes in the occurrence of serovars or specific strains in human and animal populations may follow the introduction of the strain through international travel, human migration, food, animal feed, and livestock trade (Feasey et al., 2016; Key et al., 2020; Li et al., 2022a; Li et al., 2022b; Li et al., 2021; Pulford et al., 2021; Puyvelde et al., 2019).

However, despite the very large number of publications on the occurrence and diversity of *Salmonella* within individual countries, there are surprisingly few studies on the global distribution (Ferrari et al., 2019; Gutema et al., 2019; Qin et al., 2022; Ramtahal et al., 2022; Shen et al., 2022; Sun et al., 2021; Voss-Rech et al., 2017).

Whole genome sequencing (WGS) has been increasingly used to characterize bacterial isolates, for research, outbreak detection, and surveillance. A large part of these data are shared publicly and could potentially provide novel insights into the global distribution, diversity, and transmission of *Salmonella* serovars and AMR. Thus, a decade ago it was predicted that local sequencing and global sharing of WGS data would replace conventional testing and data sharing for global surveillance of probably initially foodborne pathogens, but eventually all pathogens (Aarestrup et al., 2012; Köser et al., 2012; Quainoo et al., 2017; Rossen et al., 2018; WHO, 2018).

However, despite being used for comparison with local surveillance initiatives, there has to the best of our knowledge never been any studies evaluating the usefulness of the publicly shared data for *Salmonella* surveillance.

This study aimed to investigate the current usefulness of these publicly available data for surveillance of *Salmonella*, the distribution overview of serovars, AMR, and the most common antibiotic resistance gene (ARG) profiles.

## Materials and Methods

### Data collection and standardization

A total of 191,306 *Salmonella* WGS data and metadata were downloaded from European Nucleotide Archive (ENA) (November 17, 2020). The metadata from the NCBI Pathogen Detection project was also downloaded and merged with the ENA metadata (November 17, 2020). Combined data that did not have information on either geographical location (country), isolation source (source), or year were excluded. Duplicate data from the same source or outbreak were also excluded. We did genome quality checking with FoodQCPipeline (CGE, 2016) and species confirmation with KmerFinder v.3.2 (default setting) (Clausen et al., 2018; Hasman et al., 2014; Larsen et al., 2014).

Low-quality genomes or non-*S. enterica* were excluded. Our final data set included 47,452 isolates from 116 different countries across 7 continents (Africa, Asia, Europe, the Middle East, North America, Oceania, and South America). The sources of isolates were clustered into 11 clusters (avian, bovine, environment, feed, food, human, nut/bean, others, plant, swine, and water). The isolates represented common serovars that were 70% of all identified serovars. The duration of isolation was 1905–2020. The data obtained from single countries might not reflect the epidemiological situation of *Salmonella* in the country because the reason for submitting the data to NCBI or ENA was not considered and remains unknown. All data sets can be searched at https://microreact.org/project/tptbR3fX8fa7p5zGV5VRbu-publicly-available-salmonella.

### Genome assembly and quality filtering

Assemblies were generated by the in-house software called FoodQCPipeline. The pipeline trimmed the raw reads using bbduk2 (part of BBtools version 36.49, https://jgi.doe.gov/data-and-tools/bbtools) according to the following: (1) length of read higher or equal to 50 base pairs (bp), (2) phred score per base higher or equal to 20, and (3) adapters filtered away based on an internal database with Illumina adapters. The pipeline uses FastQC10 version 0.11.5 for fastq quality checking before and after trimming (Babraham Bioinformatics, 2016). The pipeline uses SPAdes v.3.11.0 for genomic assembly (Bankevich et al., 2012).

### *In silico* analysis

The serovar of the 47,452 assembled genomes was predicted with SISTR v.1.1.1 with default setting (Yoshida et al., 2016) and compared with the reported serovar of the ENA and NCBI metadata. In case of disagreement between SISTR predictions and the informed serovar by ENA and NCBI metadata, the results of SISTR prediction with quality checking were considered. All failures of the quality checking results were classified as unidentified (*n* = 1729). For identification of ARGs, ResFinder v.4.1 was used with the default setting, at least 60% minimum length and 90% identity, for both chromosomal point mutation and acquired ARGs (Bortolaia et al., 2020; Camacho et al., 2009; Zankari et al., 2017).

### Statistical analyses and visualization

The percentage of AMR was calculated by the number of positive-predicted AMR divided by the total number of samples (47,452). The proportion of ARGs was calculated by the number of positive-predicted ARGs divided by the total number of isolates in each continent, source, or serovar. The figures in this study were visualized in Microreact (Argimón et al., 2016).

## Results

### Information about the metadata

We found that only 25% of the isolates had sufficient epidemiological information to be useful for further analysis. The main reason for incomplete isolate data was lacking isolation year (70% of all downloaded data), whereas those isolates with missing country or source were 9.7% and 3.7%, respectively. The missing data were in isolates from humans in North America.

### The distribution of *S. enterica*

The final data set consisted of WGS data and metadata of 47,452 *S. enterica* isolates. The data were classified into 11 sources and 22 serovars. The genomes were mainly from 2011 to 2020 (87.12%), followed by 2001–2010 (10.74%). *Salmonella* genomes were isolated from human (29.10%), followed by avian (22.50%), environment (11.89%), water (9.33%), swine (6.62%), bovine (6.49%), food (4.54%), plant (1.40%), feed (1.22%), nut/bean (0.33%), and others (5.72%).

**FIG. 1.**
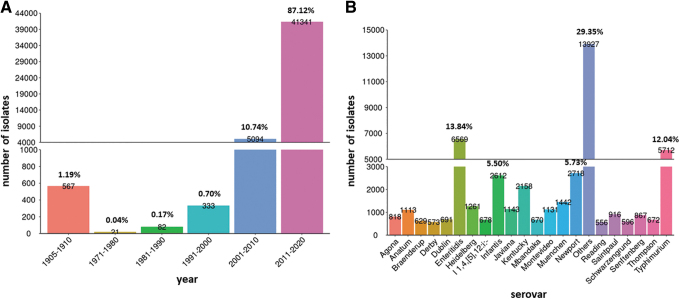
General information about distribution of *Salmonella enterica*, showing the most common years and serovars. The metadata and genomic analysis results of the 47,452 *S. enterica* isolates were categorized by year (number of isolates in each year period, *n* > 10) **(A)** and serovar **(B)**.

The top 10 common serovars were *Salmonella* Enteritidis (13.84%), *Salmonella* Typhimurium (12.04%), *Salmonella* Newport (5.73%), *Salmonella* Infantis (5.50%), *Salmonella* Kentucky (4.55%), *Salmonella* Muenchen (3.04%), *Salmonella* Heidelberg (2.66%), *Salmonella* Javiana (2.41%), *Salmonella* Montevideo (2.38%), and *Salmonella* Anatum (2.35%) (Fig. 1 and Supplementary Data S1).

**FIG. 2.**
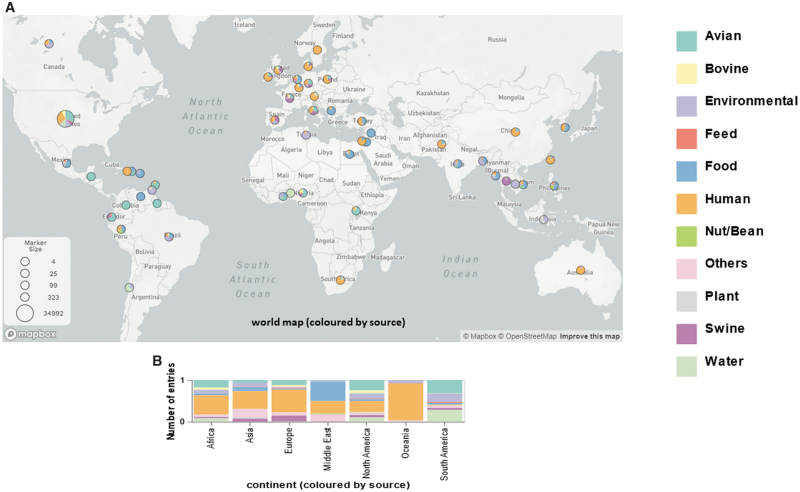
The distribution of *Salmonella enterica* in different isolation sources. The distribution of *S. enterica* was shown in the world map (number of isolates in each country, *n* > 10) **(A)**. The proportion of *S. enterica* according to isolation sources was categorized by continent **(B)**.

The relative distribution of *S. enterica* isolates from South America was almost exclusively recovered from environment, water, or avian, whereas isolates from Europe, Africa, Asia, and especially Oceania were predominantly isolated from humans. The isolates from North America were mainly isolated from human and avian samples, whereas those from the Middle East were mainly recovered from human and food samples (Fig. 2).

The distribution of *Salmonella* serovars was divided following the sources. In human samples, the most common serovars were *Salmonella* Enteritidis (8.54%), *Salmonella* Typhimurium (4.50%), and *Salmonella* Newport (2.00%). In avian samples, the most common serovars were *Salmonella* Kentucky (3.70%), *Salmonella* Enteritidis (3.16%), and *Salmonella* Infantis (2.76%). From environmental samples, the most common serovars were *Salmonella* Enteritidis (1.37%), *Salmonella* Newport (0.96%), and *Salmonella* Typhimurium (0.92%) (Supplementary Data S1).

From water samples, the most common serovars were *Salmonella* Newport (1.09%), *Salmonella* Typhimurium (0.78%), and *Salmonella* Muenchen (0.45%). From swine samples, the most common serovars were *Salmonella* Typhimurium (1.93%), *Salmonella* Derby (0.66%), and *Salmonella* Anatum (0.59%). From bovine samples, the most common serovars were *Salmonella* Dublin (1.03%), *Salmonella* Typhimurium (0.76%), and *Salmonella* Montevideo (0.68%) (Supplementary Data S1).

### The distribution of AMR

To study the distribution of AMR, we investigated the resistance in each class of antimicrobials from WGS data of *S. enterica.* The results showed the percentage of AMR for aminoglycoside (98.39%), tetracycline (23.85%), folate pathway antagonist (18.63%), β-lactam (15.78%), phenicol (7.94%), fluoroquinolone (3.36%), polymyxin (1.18%) and macrolide (0.51%) (Supplementary Data S2).

The gene *aac(6′)-Iaa* (92.22%) was commonly found in *S. enterica* genomes. The gene was also detected together with other aminoglycoside genes as *aac(3)-IV*, *aac(6′)-Iaa* resistance gene profile (2.55%), and *aac(3)-VIa*, *aac(6′)-Iaa* resistance gene profile (1.11%). The *aac(3)-IV*, *aac(6′)-Iaa* profile was predominantly harbored by *Salmonella* Infantis. The *aac(3)-VIa*, *aac(6′)-Iaa* profile was predominantly harbored by *Salmonella* Heidelberg (Supplementary Fig. S1 and Supplementary Data S3).

**FIG. 3.**
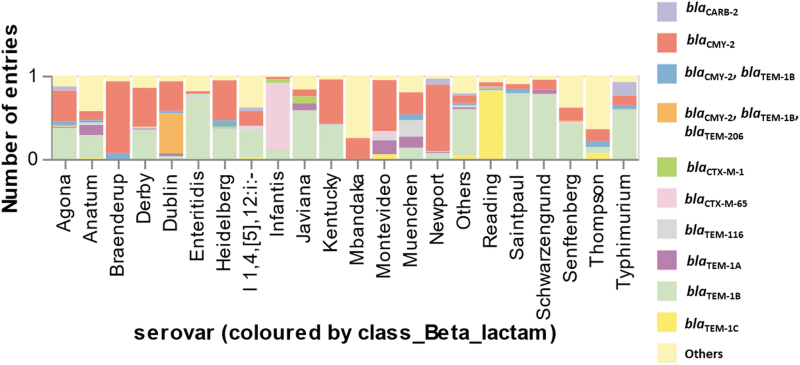
The proportion of β-lactam resistance gene profiles in *Salmonella enterica* (positive prediction = 7490 isolates) was categorized by serovar.

Common β-lactam resistance gene profiles were *bla*_TEM-1B_ (6.78%), *bla*_CMY-2_ (2.82%), and *bla*_CTX-M-65_ (1.68%). The *bla*_TEM-1B_ was predominantly harbored by *Salmonella* Heidelberg, *Salmonella* Typhimurium, and *Salmonella* Saintpaul. The *bla*_CMY-2_ was the second most common resistance profile and was predominantly harbored by *Salmonella* Heidelberg and *Salmonella* Dublin. Furthermore, *bla*_CTX-M-65_ was predominantly driven by *Salmonella* Infantis. In addition, there were other β-lactam resistance gene profiles, *bla*_CARB-2_ (1.03%) and *bla*_CMY-2_, *bla*_TEM1-B_, *bla*_TEM-206_ profile (0.54%). The *bla*_CARB-2_ was predominantly carried by *Salmonella* Typhimurium. Whereas the *bla*_CMY-2_, *bla*_TEM1-B_, *bla*_TEM-206_ profile was driven by *Salmonella* Dublin (Fig. 3 and Supplementary Data S4).

**FIG. 4.**
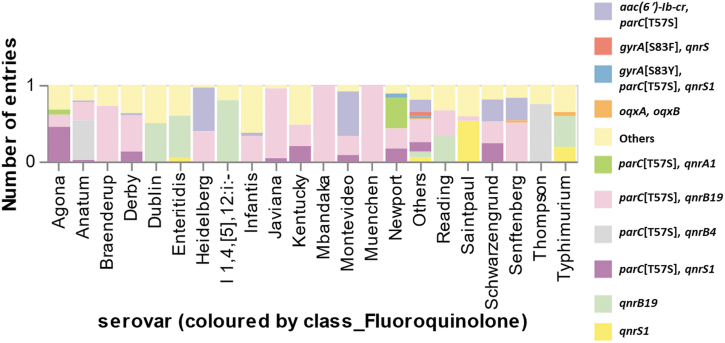
The proportion of fluoroquinolone resistance gene profiles in *Salmonella enterica* (positive prediction = 1596 isolates) was categorized by serovar.

Common fluoroquinolone resistance gene profiles were *parC*[T57S], *qnrB19* profile (0.87%); *aac(6′)-Ib-cr*, *parC*[T57S] profile (0.54%); and *qnrB19* profile (0.43%). The *parC*[T57S], *qnrB19* profile was predominantly harbored by *Salmonella* Heidelberg. The *aac(6′)-Ib-cr*, *parC*[T57S] profile was predominantly carried by *Salmonella* Heidelberg. The *qnrB19* was driven by several serovars (Fig. 4 and Supplementary Data S5).

Common folate pathway antagonist resistance gene profiles were *sul2* (8.35%); *sul1* (4.23%); and *dfrA14*, *sul1* profile (1.56%). The *sul2* was predominantly harbored in *Salmonella* Reading, *Salmonella* Typhimurium, and *Salmonella* Dublin. The *sul1* was predominantly carried by *Salmonella* Heidelberg, *Salmonella* Infantis, and *Salmonella* Derby. The *dfrA14*, *sul1* profile was predominantly driven by *Salmonella* Infantis (Supplementary Fig. S2 and Supplementary Data S6).

Common macrolide resistance gene profiles were *mph*(A) (0.39%), *mef*(B) (0.07%), and *msr*(E) (0.03%). The *mph*(A) and *mef*(B) profiles were found in several serovars. The *msr*(E) profile was predominantly harbored by *Salmonella* Agona and *Salmonella* Typhimurium (Supplementary Fig. S3 and Supplementary Data S7).

Common phenicol resistance gene profiles were *floR* (5.94%), *catA1* (0.51%), and *cmlA1* (0.45%). The *floR* profile was predominantly carried by *Salmonella* Infantis and *Salmonella* Dublin. The *catA1* profile was predominantly harbored in Dublin. The *cmlA1* profile was prominently driven by several serovars (Supplementary Fig. S4 and Supplementary Data S8).

Common polymyxin resistance gene profiles were *mcr-1.1* (0.09%), *mcr-5.1* (0.04%), and *mcr-3.1* (0.02%). The *mcr-1.1* profile was driven by several serovars. The *mcr-5.1* profile was predominantly carried by *Salmonella* Typhimurium and *Salmonella* 1,4,[5],12:i:- (Supplementary Fig. S5 and Supplementary Data S9).

Common tetracycline resistance gene profiles were *tet*(A) (12.95%), *tet*(B) (8.00%), and *tet*(G) (0.88%). The *tet*(A) profile was the most common tetracycline resistance profile predominantly harbored by *Salmonella* Derby, *Salmonella* Infantis, and *Salmonella* Dublin. The *tet*(B) profile was predominantly carried by *Salmonella* Kentucky. The *tet*(G) profile was predominantly harbored by *Salmonella* Typhimurium (Supplementary Fig. S6 and Supplementary Data S10).

### Distribution of AMR in the United States

We found the same resistance gene profiles in the United States as in other countries. Common aminoglycoside resistance gene profiles were *aac(3)-IV*, *aac(6′)-Iaa* profile (2.06%) and *aac(3)-VIa*, *aac(6′)-Iaa* profile (1.33%). Common β-lactam resistance gene profiles were *bla*_TEM-1B_ (4.33%), *bla*_CMY-2_ (3.36%), and *bla*_CTX-M-65_ (1.24%). Common fluoroquinolone resistance gene profiles were *parC*[T57S], *qnrB19* profile (0.83%); *aac(6′)-Ib-cr*, *parC*[T57S] profile (0.69%); and *qnrB19* (0.38%).

Common folate pathway antagonist resistance gene profiles were *sul2* (7.68%); *sul1* (4.22%); and *dfrA14*, *sul1* profile (1.15%). Common macrolide resistance gene profiles were *mph*(A) (0.21%), *mef*(B) (0.04%), and *msr*(E) (0.03%). The most common phenicol resistance gene profile was *floR* (5.58%). Common polymyxin resistance gene profile was *mcr-1.1* (0.003%). Common tetracycline gene resistance profiles were *tet*(A) (12.47%) and *tet*(B) (7.85%).

## Discussion

WGS has enhanced studies about genomic diversity among *Salmonella*. It is also useful for global surveillance and AMR tracking (Gupta et al., 2019). We found that the highest number of *Salmonella* genomes with sufficient epidemiological information to be useful for surveillance, and studies of global dissemination were from the United States. Although *Salmonella* genomes from 116 countries were included, however, 25% of the genomes could skew the actual percentage of AMR and ARG profile results through incomplete metadata that were excluded.

This study suggests that the global generation of WGS data and sharing of completed metadata, especially year, country, and source, are essential and challenging for a continuous global surveillance overview of *Salmonella* apart from the cost of equipment and reagents and skills of laboratory technician and bioinformatician.

*Salmonella* Enteritidis and *Salmonella* Typhimurium were the most common serovars observed among the *Salmonella* serovars that was consistent with previous studies on global or regional collections (CDC, 2018a; EFSA-ECDC, 2021; Hendriksen et al., 2011; Rodrigues et al., 2020). The important isolation sources of both serovars were avian and human samples from several continents.

This study showed that >98.00% of *S. enterica* harbored the *aac(6′)-Iaa* gene. This was consistent with the study by Srednik et al. (2021) that found that *Salmonella* Dublin isolates (100%) in the United States recovered from cattle carried *aac(6′)-Iaa* gene detected using ResFinder. Consistently, *S. enterica* isolated from duck, chicken, and pig farms and retail markets in Eastern China harbored *aac(6′)-Iaa* gene by 95.00% (Tang et al., 2022).

This gene was not reported using AMRFinderPlus because it was ubiquitously found in *Salmonella* genomes and the presence or absence of this gene did not confer aminoglycoside resistance as a cryptic gene (Feldgarden et al., 2021; Magnet et al., 1999; Ramirez and Tolmasky, 2010; Salipante and Hall, 2003). The Resistome Tracker, a tool for exploration of AMR, stress, and virulence genes, showed that common aminoglycoside resistance genes were *aph(6)-Id* and *aph(3″)-Ib* (FDA, 2022).

Common β-lactam resistance gene profiles in *S. enterica* were *bla*_TEM-1B_, *bla*_CMY-2_, *bla*_CTX-M-65_, and *bla*_CARB-2_. *Salmonella* Enteritidis and *Salmonella* Typhimurium are the important serovars related to extended-spectrum cephalosporins (ESCs) in human infections (Arlet et al., 2006). These four profiles consisted of 12.31% of the 15.78% β-lactam resistance. The *bla*_TEM-1B_ profile was dominantly harbored in *Salmonella* isolates worldwide (Eguale et al., 2017; García et al., 2019; Tang et al., 2022). A NARMS report showed that *bla*_TEM_, *bla*_CMY_, and *bla*_CARB_ were the top three β-lactam resistance genes in the United States (CDC, 2018b).

This was consistent with the results from the Resistome Tracker that showed that *bla*_TEM_ and *bla*_CMY-2_ were common β-lactam resistance genes (FDA, 2022). In addition, *bla*_CTX-M-65_ was predominantly carried by *Salmonella* Infantis. Several reports linked *bla*_CTX-M-65_ in *Salmonella* Infantis isolates in foods to those in humans (Brown et al., 2018; Granda et al., 2019; Martínez-Puchol et al., 2021). The *bla*_CARB-2_ profile was predominantly carried by *Salmonella* Typhimurium. The resistance to ESCs is a threat to public health and should be a concern (Livermore, 2012; Monte et al., 2020). Therefore, our information is useful for surveillance of β-lactam resistance in *Salmonella* especially in countries that use PCR-based detection.

Fluoroquinolone resistance usually results from a point mutation, predominantly in the conserved quinolone resistance-determining regions (QRDR i.e., *gyr*, *par*) and plasmid-mediated quinolone resistance [PMQR i.e., *qnr*, *aac(6′)-Ib-cr*, *oqx*] (Cuypers et al., 2018). Our results highlighted *parC*[T57S], *qnrB19* profile; *aac(6′)-Ib-cr*, *parC*[T57S] profile; *qnrB19* profile; *parC*[T57S], *qnrS1* profile; and *qnrS1* profile are the resistance profiles that could confer low-level resistance to fluoroquinolone (Acheampong et al., 2019; Nordmann and Poirel, 2005).

The five profiles composed 2.31% of the 3.36% fluoroquinolone resistance. The Resistome Tracker showed that *gyrA*[D87Y] and *qnrB19* were common fluoroquinolone resistance genes (FDA, 2022). A study of *Salmonella* in Brazil showed that *parC*[T57S] was the most frequent in animal-based food (Rodrigues et al., 2020). Fluoroquinolones and third-generation cephalosporins are recommended for treating invasive *Salmonella* infections or patients at risk of developing an invasive infection (Shane et al., 2017).

Common folate pathway antagonist resistance gene profiles were *sul2*; *sul1*; and *dfrA14*, *sul1*. These three profiles composed of 14.14% of the 18.63% of folate pathway antagonist resistance. The *sul2* profile was predominantly carried by *Salmonella* Typhimurium and *Salmonella* Dublin. This was consistent with the Resistome Tracker that showed that *sul2* and *sul1* were common folate pathway antagonist resistance genes (FDA, 2022). Interestingly, the *dfrA14*, *sul1* resistance gene profile was predominantly harbored by *Salmonella* Infantis.

Common macrolide resistance gene profiles found in our study were *mph*(A), *mef*(B), and *msr*(E). These profiles composed of 0.49% of the 0.51% macrolide resistance. The *mph*(A) was predominantly carried by *Salmonella* Newport. The Resistome Tracker similarly showed that *mph*(A) was the most common macrolide resistance gene (FDA, 2022).

A common phenicol resistance gene profile was *floR.* The gene consisted of 5.94% of the 7.94% phenicol resistance gene profiles. The gene was predominantly harbored by *Salmonella* Infantis and *Salmonella* Dublin. The results from the Resistome Tracker showed that *floR* was the most common phenicol resistance gene (FDA, 2022).

There are two main resistance genes displaying resistance against polymyxin, (1) alteration of polymyxin resistance gene (i.e., *pmr*) and (2) plasmid-mediated colistin resistance mechanism (i.e., *mcr*). The most common *mcr* gene in our study was *mcr-1.1* corresponding to 0.09% of 1.18% of the overall polymyxin resistance genes. Whereas *mcr-9* (0.99%) was not associated with colistin resistance in *Salmonella* and *Escherichia coli* (Tyson et al., 2020).

Common tetracycline resistance gene profiles were *tet*(A) and *tet*(B). Both profiles consisted of 20.95% of the 23.85% tetracycline resistance. The Resistome Tracker also showed that *tet*(A) and *tet*(B) were common tetracycline resistance genes (FDA, 2022). In addition, a study of *Salmonella* in the last four decades in Brazil showed that *tet*(A) was highly frequent among analyzed WGS data (Rodrigues et al., 2020).

## Conclusions

*S. enterica* can be found as a commensal organism or pathogen in animals and humans. AMR in *S. enterica* has increasingly impacted both animal and human health and remains a public health concern. Public online WGS data and shared metadata can improve global surveillance especially by supporting studies on *Salmonella* epidemiology and AMR. The results showed the distribution overview of serovars, AMR, and common ARG profiles. *Salmonella* Enteritidis and *Salmonella* Typhimurium were found on all continents and mainly recovered from avian and human samples.

Common AMR in *S. enterica* according to the class of antimicrobials was tetracycline (23.85%), folate pathway antagonist (18.63%), and β-lactam (15.78%). The most common ARG profiles in *S. enterica* were β-lactam (*bla*_TEM-1B_), fluoroquinolone (*parC*[T57S], *qnrB19*), folate pathway antagonist (*sul2*), macrolide [*mph*(A)], phenicol (*floR*), polymyxin B (*mcr-1.1*), and tetracycline [*tet*(A)]. This study showed that updating and sharing data are one of the key points for better surveillance of *Salmonella*.

## Supplementary Material

Supplemental data

Supplemental data

Supplemental data

Supplemental data

Supplemental data

Supplemental data

Supplemental data

Supplemental data

Supplemental data

Supplemental data

Supplemental data

Supplemental data

Supplemental data

Supplemental data

Supplemental data

Supplemental data

Supplemental data
