# Supplementary material for: An Overview of Antimicrobial Resistance Profiles of Publicly Available Salmonella Genomes with Sufficient Quality and Metadata
Source: Foodborne Pathog Dis. 2023 Sep 4;20(9):405–13. doi: 10.1089/fpd.2022.0080 (PMC10510693; doi:10.1089/fpd.2022.0080)
Supplement: Supplemental data [file Supp_DataS1.pdf]

SUPPLEMENTARY DATA S1. THE PERCENTAGE OF *SALMONELLA ENTERICA* DISTRIBUTION IN THIS STUDY

The percentage of *Salmonella enterica* distribution divided by serovars and isolation sources

Serovars/Sources	Human	Avian	Environmen	Water	Swine	Bovine	Others	Food	Plant	Feed	Nut/Bean	Grand Total
Agona	0.38%	0.33%	0.35%	0.04%	0.18%	0.15%	0.09%	0.10%	0.01%	0.07%	0.03%	1.72%
Anatum	0.23%	0.27%	0.24%	0.19%	0.59%	0.40%	0.18%	0.11%	0.02%	0.04%	0.07%	2.35%
Braenderup	0.38%	0.29%	0.18%	0.16%	0.01%	0.03%	0.20%	0.04%	0.02%	0.01%	0.02%	1.33%
Derby	0.07%	0.22%	0.08%	0.00%	0.66%	0.03%	0.03%	0.07%	0.00%	0.03%	0.01%	1.21%
Dublin	0.25%	0.01%	0.07%	0.01%	0.01%	1.03%	0.03%	0.03%	0.00%	0.01%	0.00%	1.46%
Enteritidis	8.54%	3.16%	1.37%	0.25%	0.02%	0.05%	0.09%	0.24%	0.00%	0.04%	0.08%	13.84%
Heidelberg	0.47%	1.57%	0.20%	0.04%	0.08%	0.09%	0.03%	0.16%	0.00%	0.01%	0.01%	2.66%
I 1,4,[5],12:i:-	0.59%	0.34%	0.07%	0.19%	0.04%	0.05%	0.04%	0.03%	0.00%	0.05%	0.04%	1.43%
Infantis	0.79%	2.76%	0.66%	0.34%	0.42%	0.13%	0.11%	0.12%	0.03%	0.13%	0.01%	5.50%
Javiana	1.09%	0.06%	0.31%	0.43%	0.00%	0.00%	0.29%	0.07%	0.14%	0.00%	0.00%	2.41%
Kentucky	0.22%	3.70%	0.18%	0.06%	0.02%	0.12%	0.06%	0.15%	0.00%	0.03%	0.00%	4.55%
Mbandaka	0.11%	0.21%	0.52%	0.07%	0.07%	0.17%	0.05%	0.16%	0.00%	0.04%	0.02%	1.41%
Montevideo	0.29%	0.16%	0.47%	0.35%	0.03%	0.68%	0.13%	0.14%	0.03%	0.05%	0.04%	2.38%
Muenchen	0.41%	0.93%	0.35%	0.45%	0.10%	0.26%	0.19%	0.21%	0.03%	0.01%	0.09%	3.04%
Newport	2.00%	0.29%	0.96%	1.09%	0.11%	0.47%	0.48%	0.19%	0.06%	0.02%	0.05%	5.73%
Others	7.57%	3.07%	3.89%	4.16%	2.11%	1.81%	2.97%	1.94%	0.92%	0.51%	0.38%	29.35%
Reading	0.13%	0.79%	0.08%	0.01%	0.03%	0.06%	0.05%	0.03%	0.00%	0.00%	0.00%	1.17%
Saintpaul	0.52%	0.66%	0.19%	0.28%	0.07%	0.02%	0.06%	0.09%	0.00%	0.01%	0.03%	1.93%
Schwarzengrund	0.09%	0.75%	0.15%	0.02%	0.04%	0.04%	0.03%	0.09%	0.01%	0.04%	0.00%	1.26%
Senftenberg	0.10%	0.39%	0.53%	0.05%	0.08%	0.08%	0.06%	0.27%	0.01%	0.05%	0.21%	1.83%
Thompson	0.36%	0.22%	0.12%	0.36%	0.00%	0.04%	0.09%	0.06%	0.08%	0.01%	0.06%	1.42%
Typhimurium	4.50%	2.29%	0.92%	0.78%	1.93%	0.76%	0.47%	0.26%	0.02%	0.06%	0.05%	12.04%
**Grand Total**	**29.10%**	**22.50%**	**11.89%**	**9.33%**	**6.62%**	**6.49%**	**5.72%**	**4.54%**	**1.40%**	**1.22%**	**1.19%**	**100.00%**

Note: The percentage was calculated by the number of isolates in each cell divided by the total number of samples (47,452).
