# Supplementary material for: An Overview of Antimicrobial Resistance Profiles of Publicly Available Salmonella Genomes with Sufficient Quality and Metadata
Source: Foodborne Pathog Dis. 2023 Sep 4;20(9):405–13. doi: 10.1089/fpd.2022.0080 (PMC10510693; doi:10.1089/fpd.2022.0080)
Supplement: Supplemental data [file Supp_DataS2.pdf]

SUPPLEMENTARY DATA S2. THE PERCENTAGE OF POSITIVE-PREDICTED AMR IN *SALMONELLA ENTERICA* IN THIS STUDY

The percentage of positive-predicted AMR in *Salmonella enterica* divided by isolation sources and antimicrobial classes

Sources/[*]Antimicrob	[1]Aminoglyc	[2]β-lactam	[3]Fluoroquin	[4]Folate path	[5]Macrolide	[6]Phenicol	[7]Polymyxin	[8]Tetracycline
Human	28.41%	4.77%	1.09%	4.79%	0.19%	1.80%	0.10%	4.56%
Avian	22.28%	4.68%	0.81%	6.12%	0.08%	1.92%	0.71%	9.87%
Environmental	11.78%	0.97%	0.24%	1.25%	0.03%	0.60%	0.05%	1.57%
Water	9.10%	0.20%	0.08%	0.35%	0.00%	0.16%	0.00%	0.44%
Swine	6.58%	2.44%	0.64%	2.87%	0.11%	1.22%	0.16%	3.55%
Bovine	6.44%	1.80%	0.17%	2.09%	0.04%	1.66%	0.03%	2.38%
Food	4.49%	0.31%	0.16%	0.45%	0.01%	0.14%	0.08%	0.60%
Plant	1.39%	0.01%	0.01%	0.01%	0.00%	0.00%	0.00%	0.03%
Feed	1.22%	0.14%	0.02%	0.17%	0.01%	0.07%	0.01%	0.21%
Nut/Bean	1.18%	0.02%	0.00%	0.02%	0.00%	0.01%	0.00%	0.02%
Others	5.52%	0.46%	0.13%	0.52%	0.04%	0.35%	0.03%	0.63%
**Grand Total**	**98.39%**	**15.78%**	**3.36%**	**18.63%**	**0.51%**	**7.94%**	**1.18%**	**23.85%**

*	Antimicrobial classes
1	Aminoglycoside
2	β-lactam
3	Fluoroquinolone
4	Folate pathway antagonist
5	Macrolide
6	Phenicol
7	Polymyxin
8	Tetracycline

Note: The percentage of AMR was calculated by the number of positive-predicted AMR in each cell divided by the total number of samples (47,452).
