# Supplementary material for: An Overview of Antimicrobial Resistance Profiles of Publicly Available Salmonella Genomes with Sufficient Quality and Metadata
Source: Foodborne Pathog Dis. 2023 Sep 4;20(9):405–13. doi: 10.1089/fpd.2022.0080 (PMC10510693; doi:10.1089/fpd.2022.0080)
Supplement: Supplemental data [file Supp_DataS3.pdf]

SUPPLEMENTARY DATA S3. THE PROPORTION (%) OF AMINOGLYCOSIDE RESISTANCE GENE PROFILES IN *SALMONELLA ENTERICA* IN THIS STUDY

The proportion (%) of aminoglycoside resistance gene profiles in *Salmonella enterica* divided by isolation sources

Sources/[*]Aminoglyc	[1]*aac(6')-Iac*	[2]*aac(3)-IV,*	[3]None	[4]*aac(3)-VIa*	[5]*aac(3)-IId,*	[6]Others	[7]*aac(6')-Iac*	[8]*aac(6')-Iac*	[9]*aac(3)-Id, (*	[10]*aac(3)-VI(*	[11]*aac(6')-Ia*	[12]*aac(6')-Ia*	Grand Total
Avian	82.80%	7.85%	1.00%	3.32%	1.55%	0.62%	0.99%	0.30%	0.44%	0.59%	0.53%	0.00%	100.00%
Bovine	96.69%	0.16%	0.78%	0.65%	0.16%	0.32%	0.00%	0.94%	0.16%	0.06%	0.03%	0.03%	100.00%
Environmental	96.55%	1.51%	0.94%	0.32%	0.25%	0.23%	0.02%	0.12%	0.00%	0.04%	0.04%	0.00%	100.00%
Feed	94.15%	1.03%	0.34%	0.34%	3.61%	0.17%	0.00%	0.00%	0.00%	0.34%	0.00%	0.00%	100.00%
Food	93.69%	0.51%	1.07%	1.02%	0.65%	0.65%	0.84%	0.14%	0.51%	0.46%	0.46%	0.00%	100.00%
Human	94.68%	0.73%	2.37%	0.44%	0.68%	0.57%	0.01%	0.12%	0.29%	0.01%	0.00%	0.08%	100.00%
Nut/Bean	98.76%	0.00%	0.88%	0.00%	0.35%	0.00%	0.00%	0.00%	0.00%	0.00%	0.00%	0.00%	100.00%
Others	94.36%	0.37%	3.50%	0.26%	0.48%	0.63%	0.00%	0.18%	0.04%	0.00%	0.00%	0.18%	100.00%
Plant	99.24%	0.00%	0.45%	0.30%	0.00%	0.00%	0.00%	0.00%	0.00%	0.00%	0.00%	0.00%	100.00%
Swine	89.78%	3.31%	0.60%	1.18%	1.50%	2.04%	0.00%	0.80%	0.16%	0.03%	0.06%	0.54%	100.00%
Water	96.18%	1.08%	2.42%	0.09%	0.07%	0.16%	0.00%	0.00%	0.00%	0.00%	0.00%	0.00%	100.00%
**Grand Total**	**92.22%**	**2.55%**	**1.61%**	**1.11%**	**0.80%**	**0.57%**	**0.27%**	**0.25%**	**0.23%**	**0.17%**	**0.15%**	**0.07%**	**100.00%**

*	Aminoglycoside resistance gene profiles
1	*aac(6')-Iaa;*
2	*aac(3)-IV, aac(6')-Iaa;*
3	None;
4	*aac(3)-VIa, aac(6')-Iaa;*
5	*aac(3)-IId, aac(6')-Iaa;*
6	Others;
7	*aac(6')-Iaa, aac(6')-Ib3, aac(6')-Ib-cr, grdA;*
8	*aac(6')-Iaa, ant(2'')-Ia;*
9	*aac(3)-Id, aac(6')-Iaa;*
10	*aac(3)-VIa, aac(6')-Iaa, aac(6')-Ib3, aac(6')-Ib-cr, grdA;*
11	*aac(6')-Iaa, grdA;*
12	*aac(6')-Iaa, aac(6')-IIc;*

The proportion (%) of aminoglycoside resistance gene profiles in *Salmonella enterica* divided by serovars

Serovars/[*]Aminogly	[1]*aac(6')-Iac*	[2]*aac(3)-IV,*	[3]None	[4]*aac(3)-VIa*	[5]*aac(3)-IId,*	[6]Others	[7]*aac(6')-Iac*	[8]*aac(6')-Iac*	[9]*aac(3)-Id, (*	[10]*aac(3)-VI(*	[11]*aac(6')-Ia*	[12]*aac(6')-Ia*	Grand Total
Agona	93.03%	1.22%	0.49%	1.47%	1.22%	2.32%	0.00%	0.24%	0.00%	0.00%	0.00%	0.00%	100.00%
Anatum	96.95%	1.26%	0.63%	0.27%	0.18%	0.54%	0.00%	0.00%	0.00%	0.00%	0.00%	0.18%	100.00%
Braenderup	96.82%	0.00%	0.95%	1.75%	0.00%	0.32%	0.00%	0.16%	0.00%	0.00%	0.00%	0.00%	100.00%
Derby	93.19%	1.40%	0.00%	4.71%	0.35%	0.35%	0.00%	0.00%	0.00%	0.00%	0.00%	0.00%	100.00%
Dublin	94.36%	0.00%	1.30%	0.00%	0.29%	0.00%	0.00%	4.05%	0.00%	0.00%	0.00%	0.00%	100.00%
Enteritidis	97.35%	0.08%	2.10%	0.15%	0.15%	0.17%	0.00%	0.00%	0.00%	0.00%	0.00%	0.00%	100.00%
Heidelberg	69.55%	0.56%	1.27%	12.61%	2.46%	3.01%	2.22%	2.22%	0.00%	5.63%	0.32%	0.16%	100.00%
I 1,4,[5],12:i:-	93.81%	0.29%	1.33%	1.47%	3.10%	0.00%	0.00%	0.00%	0.00%	0.00%	0.00%	0.00%	100.00%
Infantis	60.41%	38.17%	0.46%	0.57%	0.00%	0.23%	0.00%	0.15%	0.00%	0.00%	0.00%	0.00%	100.00%
Javiana	98.78%	0.00%	1.05%	0.00%	0.00%	0.09%	0.00%	0.00%	0.00%	0.00%	0.00%	0.09%	100.00%
Kentucky	93.79%	0.05%	0.05%	1.25%	0.09%	0.32%	0.00%	0.00%	4.45%	0.00%	0.00%	0.00%	100.00%
Mbandaka	98.51%	0.30%	0.15%	0.90%	0.00%	0.00%	0.00%	0.15%	0.00%	0.00%	0.00%	0.00%	100.00%
Montevideo	97.61%	0.18%	0.53%	0.27%	0.09%	0.35%	0.27%	0.71%	0.00%	0.00%	0.00%	0.00%	100.00%
Muenchen	98.47%	0.07%	0.90%	0.00%	0.00%	0.07%	0.00%	0.00%	0.00%	0.00%	0.49%	0.00%	100.00%
Newport	98.05%	0.11%	0.96%	0.55%	0.15%	0.07%	0.00%	0.04%	0.04%	0.00%	0.04%	0.00%	100.00%
Others	94.36%	0.33%	2.73%	0.58%	0.39%	0.49%	0.55%	0.11%	0.06%	0.08%	0.27%	0.04%	100.00%
Reading	96.58%	0.00%	0.54%	2.52%	0.18%	0.18%	0.00%	0.00%	0.00%	0.00%	0.00%	0.00%	100.00%
Saintpaul	80.24%	1.86%	0.87%	2.18%	14.30%	0.44%	0.00%	0.11%	0.00%	0.00%	0.00%	0.00%	100.00%
Schwarzengrund	92.62%	0.67%	0.34%	1.51%	2.68%	2.18%	0.00%	0.00%	0.00%	0.00%	0.00%	0.00%	100.00%
Senftenberg	89.16%	0.12%	2.42%	1.61%	0.00%	0.92%	2.19%	1.04%	0.00%	0.00%	2.54%	0.00%	100.00%
Thompson	96.28%	0.00%	2.38%	0.00%	0.00%	1.19%	0.00%	0.15%	0.00%	0.00%	0.00%	0.00%	100.00%
Typhimurium	91.93%	1.54%	1.31%	1.61%	1.58%	1.23%	0.00%	0.32%	0.05%	0.00%	0.02%	0.42%	100.00%
**Grand Total**	**92.22%**	**2.55%**	**1.61%**	**1.11%**	**0.80%**	**0.57%**	**0.27%**	**0.25%**	**0.23%**	**0.17%**	**0.15%**	**0.07%**	**100.00%**

Note: The percentage (proportion) of ARGs was calculated by the number of positive-predicted ARGs (each cell) divided by the total number of isolates (each row)
