# Supplementary material for: An Overview of Antimicrobial Resistance Profiles of Publicly Available Salmonella Genomes with Sufficient Quality and Metadata
Source: Foodborne Pathog Dis. 2023 Sep 4;20(9):405–13. doi: 10.1089/fpd.2022.0080 (PMC10510693; doi:10.1089/fpd.2022.0080)
Supplement: Supplemental data [file Supp_DataS4.pdf]

SUPPLEMENTARY DATA S4. THE PROPORTION (%) OF β-LACTAM RESISTANCE GENE PROFILES IN *SALMONELLA ENTERICA* IN THIS STUDY

The proportion (%) of β-lactam resistance gene profiles in *Salmonella enterica* divided by isolation sources

Sources/[*]Beta-lactar	[1]None	[2]$bla_{\text{TEM-1B}}$	[3]$bla_{\text{CMY-2}}$	[4]$bla_{\text{CTX-M-65}}$	[5]Others	[6]$bla_{\text{CARB-2}}$	[7]$bla_{\text{CMY-2}}$, bl	[8]$bla_{\text{CMY-2}}$, bl	[9]$bla_{\text{TEM-1C}}$	[10]$bla_{\text{TEM-1A}}$	[11]$bla_{\text{TEM-116}}$	[12]$bla_{\text{CTX-M-1}}$	Grand Total
Avian	79.21%	6.59%	3.64%	5.94%	1.51%	0.22%	0.03%	0.67%	1.68%	0.22%	0.02%	0.27%	100.00%
Bovine	72.26%	3.25%	11.85%	0.03%	1.92%	2.24%	6.79%	1.07%	0.03%	0.52%	0.03%	0.00%	100.00%
Environmental	91.83%	2.85%	2.32%	1.08%	0.50%	0.44%	0.27%	0.18%	0.30%	0.14%	0.09%	0.00%	100.00%
Feed	88.30%	1.89%	2.07%	0.86%	3.96%	2.07%	0.00%	0.00%	0.00%	0.69%	0.17%	0.00%	100.00%
Food	93.27%	2.79%	1.86%	0.19%	0.46%	0.60%	0.09%	0.05%	0.19%	0.14%	0.37%	0.00%	100.00%
Human	83.62%	10.17%	1.56%	0.39%	2.22%	1.17%	0.14%	0.25%	0.09%	0.05%	0.22%	0.12%	100.00%
Nut/Bean	98.41%	0.53%	0.71%	0.00%	0.00%	0.00%	0.00%	0.00%	0.00%	0.18%	0.18%	0.00%	100.00%
Others	92.00%	2.80%	2.03%	0.15%	1.36%	1.40%	0.00%	0.04%	0.07%	0.15%	0.00%	0.00%	100.00%
Plant	99.55%	0.00%	0.00%	0.00%	0.15%	0.30%	0.00%	0.00%	0.00%	0.00%	0.00%	0.00%	100.00%
Swine	63.21%	22.02%	3.47%	0.19%	3.50%	4.46%	0.22%	2.45%	0.00%	0.29%	0.06%	0.13%	100.00%
Water	97.85%	0.16%	0.36%	0.63%	0.70%	0.16%	0.00%	0.00%	0.02%	0.09%	0.02%	0.00%	100.00%
**Grand Total**	**84.22%**	**6.78%**	**2.82%**	**1.68%**	**1.61%**	**1.03%**	**0.54%**	**0.48%**	**0.46%**	**0.17%**	**0.11%**	**0.10%**	**100.00%**

*	β-lactam resistance gene profiles
1	None;
2	$bla_{\text{TEM-1B}}$;
3	$bla_{\text{CMY-2}}$;
4	$bla_{\text{CTX-M-65}}$;
5	Others;
6	$bla_{\text{CARB-2}}$;
7	$bla_{\text{CMY-2}}$, $bla_{\text{TEM-1B}}$, $bla_{\text{TEM-206}}$;
8	$bla_{\text{CMY-2}}$, $bla_{\text{TEM-1B}}$;
9	$bla_{\text{TEM-1C}}$;
10	$bla_{\text{TEM-1A}}$;
11	$bla_{\text{TEM-116}}$;
12	$bla_{\text{CTX-M-1}}$;

The proportion (%) of β-lactam resistance gene profiles in *Salmonella enterica* divided by serovars

Serovars/[*]Beta-lacta	[1]None	[2]$bla_{\text{TEM-1B}}$	[3]$bla_{\text{CMY-2}}$	[4]$bla_{\text{CTX-M-65}}$	[5]Others	[6]$bla_{\text{CARB-2}}$	[7]$bla_{\text{CMY-2}}$, bl	[8]$bla_{\text{CMY-2}}$, bl	[9]$bla_{\text{TEM-1C}}$	[10]$bla_{\text{TEM-1A}}$	[11]$bla_{\text{TEM-116}}$	[12]$bla_{\text{CTX-M-1}}$	Grand Total
Agona	84.96%	5.62%	5.62%	0.00%	1.96%	0.73%	0.24%	0.61%	0.00%	0.12%	0.12%	0.00%	100.00%
Anatum	90.93%	2.52%	0.99%	0.00%	3.86%	0.00%	0.00%	0.27%	0.09%	1.08%	0.27%	0.00%	100.00%
Braenderup	97.62%	0.00%	2.07%	0.00%	0.16%	0.00%	0.00%	0.16%	0.00%	0.00%	0.00%	0.00%	100.00%
Derby	84.64%	5.41%	7.16%	0.00%	2.27%	0.00%	0.00%	0.00%	0.00%	0.17%	0.35%	0.00%	100.00%
Dublin	27.06%	2.32%	26.05%	0.00%	4.92%	0.00%	35.02%	2.17%	0.00%	2.46%	0.00%	0.00%	100.00%
Enteritidis	92.43%	5.59%	0.23%	0.00%	1.42%	0.00%	0.00%	0.03%	0.02%	0.00%	0.27%	0.02%	100.00%
Heidelberg	71.77%	10.39%	13.56%	0.00%	1.59%	0.00%	0.00%	2.14%	0.00%	0.24%	0.00%	0.32%	100.00%
I 1,4,[5],12:i:-	89.97%	3.24%	1.77%	0.29%	3.83%	0.44%	0.00%	0.00%	0.15%	0.00%	0.29%	0.00%	100.00%
Infantis	61.94%	4.17%	1.00%	30.32%	0.57%	0.04%	0.00%	0.27%	0.00%	0.11%	0.11%	1.45%	100.00%
Javiana	98.95%	0.61%	0.09%	0.00%	0.17%	0.00%	0.00%	0.00%	0.00%	0.09%	0.00%	0.09%	100.00%
Kentucky	90.87%	3.75%	4.87%	0.00%	0.42%	0.00%	0.00%	0.00%	0.00%	0.05%	0.05%	0.00%	100.00%
Mbandaka	99.40%	0.00%	0.15%	0.00%	0.45%	0.00%	0.00%	0.00%	0.00%	0.00%	0.00%	0.00%	100.00%
Montevideo	98.41%	0.00%	0.97%	0.09%	0.09%	0.00%	0.00%	0.00%	0.09%	0.27%	0.09%	0.00%	100.00%
Muenchen	98.96%	0.14%	0.28%	0.00%	0.21%	0.00%	0.00%	0.07%	0.00%	0.14%	0.21%	0.00%	100.00%
Newport	86.87%	0.92%	10.52%	0.00%	0.48%	0.92%	0.00%	0.07%	0.00%	0.15%	0.07%	0.00%	100.00%
Others	93.71%	3.50%	0.56%	0.00%	1.34%	0.17%	0.04%	0.17%	0.27%	0.13%	0.09%	0.03%	100.00%
Reading	61.69%	0.72%	1.98%	0.00%	3.06%	0.00%	0.54%	0.18%	31.12%	0.54%	0.00%	0.18%	100.00%
Saintpaul	63.86%	28.49%	1.97%	0.00%	3.60%	0.11%	0.00%	1.97%	0.00%	0.00%	0.00%	0.00%	100.00%
Schwarzengrund	93.12%	5.37%	0.84%	0.00%	0.34%	0.00%	0.00%	0.00%	0.00%	0.34%	0.00%	0.00%	100.00%
Senftenberg	94.00%	2.54%	0.92%	0.12%	2.31%	0.00%	0.12%	0.00%	0.00%	0.00%	0.00%	0.00%	100.00%
Thompson	97.92%	0.15%	0.30%	0.00%	1.34%	0.00%	0.00%	0.15%	0.15%	0.00%	0.00%	0.00%	100.00%
Typhimurium	54.24%	27.08%	5.11%	0.02%	3.62%	7.53%	0.04%	2.14%	0.04%	0.16%	0.04%	0.00%	100.00%
**Grand Total**	**84.22%**	**6.78%**	**2.82%**	**1.68%**	**1.61%**	**1.03%**	**0.54%**	**0.48%**	**0.46%**	**0.17%**	**0.11%**	**0.10%**	**100.00%**

Note: The percentage (proportion) of ARGs was calculated by the number of positive-predicted ARGs (each cell) divided by the total number of isolates (each row)
