# Supplementary material for: An Overview of Antimicrobial Resistance Profiles of Publicly Available Salmonella Genomes with Sufficient Quality and Metadata
Source: Foodborne Pathog Dis. 2023 Sep 4;20(9):405–13. doi: 10.1089/fpd.2022.0080 (PMC10510693; doi:10.1089/fpd.2022.0080)
Supplement: Supplemental data [file Supp_DataS5.pdf]

SUPPLEMENTARY DATA S5. THE PROPORTION (%) OF FLUOROQUINOLONE RESISTANCE GENE PROFILES IN *SALMONELLA ENTERICA* IN THIS STUDY

The proportion (%) of fluoroquinolone resistance gene profiles in *Salmonella enterica* divided by isolation sources

**Sources/[*]Fluoroquin**	**[1]None**	**[2]*parC*[T57S**	**[3]Others**	**[4]*aac(6')-Ib-***	**[5]*qnrB19***	**[6]*parC* [T57S**	**[7]*qnrS1***	**[8]*parC*[T57S**	**[9]*gyrA*[S83F**	**[10]*parC*[T57S**	**[11]*oqxA, oqx***	**[12]*gyrA*[S83Y**	**Grand Total**
Avian	96.40%	0.45%	0.51%	1.78%	0.37%	0.20%	0.06%	0.11%	0.00%	0.00%	0.06%	0.07%	100.00%
Bovine	97.40%	1.62%	0.29%	0.06%	0.19%	0.23%	0.00%	0.00%	0.00%	0.19%	0.00%	0.00%	100.00%
Environmental	97.98%	1.03%	0.25%	0.18%	0.18%	0.39%	0.00%	0.00%	0.00%	0.00%	0.00%	0.00%	100.00%
Feed	98.28%	0.69%	0.17%	0.52%	0.00%	0.00%	0.00%	0.00%	0.00%	0.34%	0.00%	0.00%	100.00%
Food	96.43%	1.11%	0.28%	1.72%	0.09%	0.19%	0.00%	0.00%	0.00%	0.05%	0.14%	0.00%	100.00%
Human	96.25%	0.65%	1.07%	0.07%	0.68%	0.29%	0.39%	0.11%	0.22%	0.14%	0.09%	0.04%	100.00%
Nut/Bean	99.65%	0.18%	0.00%	0.00%	0.00%	0.00%	0.00%	0.00%	0.00%	0.00%	0.00%	0.18%	100.00%
Others	97.68%	0.41%	0.88%	0.11%	0.00%	0.52%	0.15%	0.15%	0.00%	0.00%	0.04%	0.07%	100.00%
Plant	99.24%	0.60%	0.00%	0.00%	0.00%	0.00%	0.15%	0.00%	0.00%	0.00%	0.00%	0.00%	100.00%
Swine	90.32%	3.41%	2.61%	0.10%	1.69%	0.29%	1.21%	0.10%	0.00%	0.00%	0.10%	0.19%	100.00%
Water	99.12%	0.38%	0.47%	0.00%	0.00%	0.02%	0.00%	0.00%	0.00%	0.00%	0.00%	0.00%	100.00%
**Grand Total**	**96.64%**	**0.87%**	**0.76%**	**0.54%**	**0.43%**	**0.25%**	**0.22%**	**0.07%**	**0.07%**	**0.06%**	**0.05%**	**0.05%**	**100.00%**

*	**Fluoroquinolone resistance gene profiles**
1	None;
2	*parC*[T57S], *qnrB19*;
3	Others;
4	*aac(6')-Ib-cr*, *parC*[T57S];
5	*qnrB19*;
6	*parC*[T57S], *qnrS1*;
7	*qnrS1*;
8	*parC*[T57S], *qnrB4*;
9	*gyrA*[S83F], *qnrS*;
10	*parC*[T57S], qnrA1;
11	*oqxA* , *oqxB* ;
12	*gyrA*[S83Y], *parC*[T57S], *qnrS1*;

The proportion (%) of fluoroquinolone resistance gene profiles in *Salmonella enterica* divided by serovars

**Serovars/[*]Fluoroqui**	**[1]None**	**[2]*parC*[T57S**	**[3]Others**	**[4]*aac(6')-Ib-***	**[5]*qnrB19***	**[6]*parC* [T57S**	**[7]*qnrS1***	**[8]*parC*[T57S**	**[9]*gyrA*[S83F**	**[10]*parC*[T57S**	**[11]*oqxA, oqx***	**[12]*gyrA*[S83Y**	**Grand Total**
Agona	96.21%	0.61%	1.22%	0.00%	0.00%	1.71%	0.00%	0.00%	0.00%	0.24%	0.00%	0.00%	100.00%
Anatum	95.15%	1.17%	0.99%	0.09%	0.00%	0.09%	0.00%	2.52%	0.00%	0.00%	0.00%	0.00%	100.00%
Braenderup	98.25%	1.27%	0.48%	0.00%	0.00%	0.00%	0.00%	0.00%	0.00%	0.00%	0.00%	0.00%	100.00%
Derby	93.37%	3.14%	2.44%	0.17%	0.00%	0.87%	0.00%	0.00%	0.00%	0.00%	0.00%	0.00%	100.00%
Dublin	99.71%	0.00%	0.14%	0.00%	0.14%	0.00%	0.00%	0.00%	0.00%	0.00%	0.00%	0.00%	100.00%
Enteritidis	99.06%	0.00%	0.38%	0.00%	0.52%	0.00%	0.05%	0.00%	0.00%	0.00%	0.00%	0.00%	100.00%
Heidelberg	83.19%	6.66%	0.56%	9.60%	0.00%	0.00%	0.00%	0.00%	0.00%	0.00%	0.00%	0.00%	100.00%
I 1,4,[5],12:i:-	99.26%	0.00%	0.15%	0.00%	0.59%	0.00%	0.00%	0.00%	0.00%	0.00%	0.00%	0.00%	100.00%
Infantis	99.08%	0.31%	0.57%	0.04%	0.00%	0.00%	0.00%	0.00%	0.00%	0.00%	0.00%	0.00%	100.00%
Javiana	97.99%	1.84%	0.09%	0.00%	0.00%	0.09%	0.00%	0.00%	0.00%	0.00%	0.00%	0.00%	100.00%
Kentucky	98.84%	0.32%	0.60%	0.00%	0.00%	0.23%	0.00%	0.00%	0.00%	0.00%	0.00%	0.00%	100.00%
Mbandaka	99.85%	0.15%	0.00%	0.00%	0.00%	0.00%	0.00%	0.00%	0.00%	0.00%	0.00%	0.00%	100.00%
Montevideo	98.94%	0.27%	0.09%	0.62%	0.00%	0.09%	0.00%	0.00%	0.00%	0.00%	0.00%	0.00%	100.00%
Muenchen	99.24%	0.76%	0.00%	0.00%	0.00%	0.00%	0.00%	0.00%	0.00%	0.00%	0.00%	0.00%	100.00%
Newport	98.05%	0.52%	0.22%	0.00%	0.00%	0.33%	0.00%	0.00%	0.00%	0.77%	0.00%	0.11%	100.00%
Others	95.55%	1.29%	0.86%	0.73%	0.33%	0.55%	0.24%	0.00%	0.22%	0.04%	0.05%	0.14%	100.00%
Reading	99.46%	0.18%	0.18%	0.00%	0.18%	0.00%	0.00%	0.00%	0.00%	0.00%	0.00%	0.00%	100.00%
Saintpaul	98.14%	0.11%	0.76%	0.00%	0.00%	0.00%	0.98%	0.00%	0.00%	0.00%	0.00%	0.00%	100.00%
Schwarzengrund	96.48%	1.01%	0.67%	1.01%	0.00%	0.84%	0.00%	0.00%	0.00%	0.00%	0.00%	0.00%	100.00%
Senftenberg	92.50%	3.81%	1.27%	2.19%	0.00%	0.00%	0.00%	0.00%	0.00%	0.00%	0.23%	0.00%	100.00%
Thompson	98.81%	0.00%	0.30%	0.00%	0.00%	0.00%	0.00%	0.89%	0.00%	0.00%	0.00%	0.00%	100.00%
Typhimurium	94.78%	0.00%	1.86%	0.00%	2.08%	0.00%	1.00%	0.00%	0.00%	0.00%	0.28%	0.00%	100.00%
**Grand Total**	**96.64%**	**0.87%**	**0.76%**	**0.54%**	**0.43%**	**0.25%**	**0.22%**	**0.07%**	**0.07%**	**0.06%**	**0.05%**	**0.05%**	**100.00%**

Note: The percentage (proportion) of ARGs was calculated by the number of positive-predicted ARGs (each cell) divided by the total number of isolates (each row)
