# Supplementary material for: An Overview of Antimicrobial Resistance Profiles of Publicly Available Salmonella Genomes with Sufficient Quality and Metadata
Source: Foodborne Pathog Dis. 2023 Sep 4;20(9):405–13. doi: 10.1089/fpd.2022.0080 (PMC10510693; doi:10.1089/fpd.2022.0080)
Supplement: Supplemental data [file Supp_DataS6.pdf]

SUPPLEMENTARY DATA S6. THE PROPORTION (%) OF FOLATE PATHWAY ANTAGONIST RESISTANCE GENE PROFILES IN *SALMONELLA ENTERICA* IN THIS STUDY

The proportion (%) of folate pathway antagonist resistance gene profiles in *Salmonella enterica* divided by isolation sources

Sources/[*]Folate path	[1]None	[2]*sul2*	[3]*sul1*	[4]Others	[5]*dfrA14, su*	[6]*sul1, sul2*	[7]*dfrA7, sul1*	[8]*dfrA1, sul1*	[9]*dfrA14, su*	[10]*dfrA12, su*	[11]dfrA34, su	[12]*dfrA14*	Grand Total
Avian	72.82%	9.49%	8.66%	2.11%	5.06%	0.76%	0.01%	0.15%	0.34%	0.14%	0.00%	0.47%	100.00%
Bovine	67.81%	22.60%	3.90%	1.33%	0.06%	1.59%	0.00%	0.65%	0.06%	0.91%	1.07%	0.00%	100.00%
Environmental	89.53%	5.10%	2.14%	1.33%	1.06%	0.28%	0.02%	0.07%	0.18%	0.04%	0.23%	0.02%	100.00%
Feed	85.89%	3.79%	4.65%	3.96%	0.34%	0.34%	0.00%	0.69%	0.00%	0.17%	0.00%	0.17%	100.00%
Food	90.11%	3.53%	3.76%	1.25%	0.42%	0.37%	0.00%	0.19%	0.23%	0.00%	0.09%	0.05%	100.00%
Human	83.55%	8.34%	2.40%	2.82%	0.45%	0.29%	1.00%	0.41%	0.22%	0.17%	0.20%	0.17%	100.00%
Nut/Bean	98.59%	0.88%	0.00%	0.18%	0.00%	0.00%	0.00%	0.00%	0.00%	0.35%	0.00%	0.00%	100.00%
Others	90.93%	3.69%	2.10%	2.06%	0.29%	0.26%	0.00%	0.07%	0.18%	0.26%	0.04%	0.11%	100.00%
Plant	99.40%	0.00%	0.60%	0.00%	0.00%	0.00%	0.00%	0.00%	0.00%	0.00%	0.00%	0.00%	100.00%
Swine	56.62%	17.50%	9.87%	11.78%	0.16%	1.59%	0.00%	0.32%	0.64%	0.70%	0.64%	0.19%	100.00%
Water	96.20%	1.33%	0.75%	0.45%	1.17%	0.05%	0.00%	0.00%	0.00%	0.00%	0.00%	0.05%	100.00%
**Grand Total**	**81.37%**	**8.35%**	**4.23%**	**2.59%**	**1.56%**	**0.54%**	**0.30%**	**0.25%**	**0.23%**	**0.21%**	**0.20%**	**0.18%**	**100.00%**

*	Folate pathway antagonist resistance gene profiles
1	None;
2	*sul2*;
3	*sul1*;
4	Others;
5	*dfrA14, sul1;*
6	*sul1, sul2;*
7	*dfrA7, sul1, sul2;*
8	*dfrA1, sul1;*
9	*dfrA14, sul2;*
10	*dfrA12, sul1, sul2;*
11	*dfrA34, sul1, sul2;*
12	*dfrA14;*

The proportion (%) of folate pathway antagonist resistance gene profiles in *Salmonella enterica* divided by serovars

Serovars/[*]Folate pat	[1]None	[2]*sul2*	[3]*sul1*	[4]Others	[5]*dfrA14, su*	[6]*sul1, sul2*	[7]*dfrA7, sul1*	[8]*dfrA1, sul1*	[9]*dfrA14, su*	[10]*dfrA12, su*	[11]dfrA34, su	[12]*dfrA14*	Grand Total
Agona	75.55%	7.58%	6.97%	4.77%	0.00%	0.73%	0.00%	0.00%	1.22%	0.61%	1.96%	0.61%	100.00%
Anatum	91.28%	1.71%	0.45%	5.93%	0.00%	0.09%	0.00%	0.00%	0.00%	0.54%	0.00%	0.00%	100.00%
Braenderup	97.30%	0.32%	1.75%	0.64%	0.00%	0.00%	0.00%	0.00%	0.00%	0.00%	0.00%	0.00%	100.00%
Derby	59.16%	4.36%	30.02%	4.89%	0.00%	0.70%	0.00%	0.17%	0.00%	0.52%	0.00%	0.17%	100.00%
Dublin	22.72%	73.23%	2.17%	0.14%	0.00%	1.45%	0.00%	0.14%	0.00%	0.14%	0.00%	0.00%	100.00%
Enteritidis	94.49%	4.23%	0.14%	0.75%	0.00%	0.11%	0.00%	0.23%	0.06%	0.00%	0.00%	0.00%	100.00%
Heidelberg	67.57%	3.33%	19.35%	1.90%	0.00%	1.43%	0.16%	0.00%	0.24%	0.32%	5.71%	0.00%	100.00%
I 1,4,[5],12:i:-	91.89%	2.06%	2.06%	3.54%	0.00%	0.15%	0.00%	0.00%	0.00%	0.29%	0.00%	0.00%	100.00%
Infantis	40.39%	0.31%	23.74%	4.25%	28.33%	0.46%	0.00%	0.42%	0.00%	0.00%	0.00%	2.11%	100.00%
Javiana	99.65%	0.17%	0.00%	0.17%	0.00%	0.00%	0.00%	0.00%	0.00%	0.00%	0.00%	0.00%	100.00%
Kentucky	91.94%	0.97%	5.33%	0.88%	0.00%	0.00%	0.00%	0.05%	0.83%	0.00%	0.00%	0.00%	100.00%
Mbandaka	94.33%	0.15%	2.54%	2.84%	0.00%	0.15%	0.00%	0.00%	0.00%	0.00%	0.00%	0.00%	100.00%
Montevideo	97.52%	1.15%	0.44%	0.44%	0.00%	0.27%	0.00%	0.09%	0.00%	0.09%	0.00%	0.00%	100.00%
Muenchen	86.75%	12.90%	0.00%	0.21%	0.00%	0.00%	0.00%	0.00%	0.14%	0.00%	0.00%	0.00%	100.00%
Newport	86.64%	10.85%	0.33%	0.37%	0.00%	0.22%	0.00%	1.10%	0.04%	0.37%	0.07%	0.00%	100.00%
Others	92.46%	1.65%	1.03%	2.89%	0.00%	0.17%	0.99%	0.22%	0.26%	0.12%	0.03%	0.17%	100.00%
Reading	69.42%	24.82%	1.80%	0.72%	0.00%	2.34%	0.00%	0.00%	0.90%	0.00%	0.00%	0.00%	100.00%
Saintpaul	94.00%	1.09%	2.29%	0.76%	0.00%	0.66%	0.00%	0.00%	0.87%	0.33%	0.00%	0.00%	100.00%
Schwarzengrund	87.58%	8.56%	1.01%	1.68%	0.00%	0.34%	0.00%	0.00%	0.00%	0.84%	0.00%	0.00%	100.00%
Senftenberg	92.85%	1.50%	2.54%	1.96%	0.00%	0.12%	0.00%	0.69%	0.12%	0.12%	0.12%	0.00%	100.00%
Thompson	98.07%	0.15%	0.15%	1.64%	0.00%	0.00%	0.00%	0.00%	0.00%	0.00%	0.00%	0.00%	100.00%
Typhimurium	44.80%	35.77%	8.96%	6.53%	0.00%	2.45%	0.00%	0.35%	0.35%	0.74%	0.02%	0.04%	100.00%
**Grand Total**	**81.37%**	**8.35%**	**4.23%**	**2.59%**	**1.56%**	**0.54%**	**0.30%**	**0.25%**	**0.23%**	**0.21%**	**0.20%**	**0.18%**	**100.00%**

Note: The percentage (proportion) of ARGs was calculated by the number of positive-predicted ARGs (each cell) divided by the total number of isolates (each row)
