# Supplementary material for: An Overview of Antimicrobial Resistance Profiles of Publicly Available Salmonella Genomes with Sufficient Quality and Metadata
Source: Foodborne Pathog Dis. 2023 Sep 4;20(9):405–13. doi: 10.1089/fpd.2022.0080 (PMC10510693; doi:10.1089/fpd.2022.0080)
Supplement: Supplemental data [file Supp_DataS7.pdf]

SUPPLEMENTARY DATA S7. THE PROPORTION (%) OF MACROLIDE RESISTANCE GENE PROFILES IN *SALMONELLA ENTERICA* IN THIS STUDY

The proportion (%) of macrolide resistance gene profiles in *Salmonella enterica* divided by isolation sources

Sources/[*]Macrolide r	[1]None	[2]*mph*(A)	[3]*mef*(B)	[4]*msr*(E)	[5]*mph*(A), *m*	[6]*mef*(B), *mp*	[7]*mef*(A), *ms*	[8]*mef*(B), *ms*	Grand Total
Avian	99.64%	0.27%	0.07%	0.00%	0.00%	0.01%	0.00%	0.00%	100.00%
Bovine	99.42%	0.49%	0.06%	0.00%	0.00%	0.00%	0.03%	0.00%	100.00%
Environmental	99.77%	0.07%	0.16%	0.00%	0.00%	0.00%	0.00%	0.00%	100.00%
Feed	99.48%	0.52%	0.00%	0.00%	0.00%	0.00%	0.00%	0.00%	100.00%
Food	99.77%	0.23%	0.00%	0.00%	0.00%	0.00%	0.00%	0.00%	100.00%
Human	99.36%	0.56%	0.02%	0.03%	0.02%	0.00%	0.00%	0.00%	100.00%
Nut/Bean	100.00%	0.00%	0.00%	0.00%	0.00%	0.00%	0.00%	0.00%	100.00%
Others	99.23%	0.74%	0.04%	0.00%	0.00%	0.00%	0.00%	0.00%	100.00%
Plant	100.00%	0.00%	0.00%	0.00%	0.00%	0.00%	0.00%	0.00%	100.00%
Swine	98.28%	0.99%	0.38%	0.25%	0.06%	0.00%	0.00%	0.03%	100.00%
Water	100.00%	0.00%	0.00%	0.00%	0.00%	0.00%	0.00%	0.00%	100.00%
**Grand Total**	**99.49%**	**0.39%**	**0.07%**	**0.03%**	**0.01%**	**0.00%**	**0.00%**	**0.00%**	**100.00%**

*	Macrolide resistance gene profiles
1	None;
2	*mph* (A);
3	*mef* (B);
4	*msr* (E);
5	*mph* (A), *msr* (E);
6	mef(B), *mph* (A);
7	*mef* (A), *msr* (D);
8	*mef* (B), *msr* (E);

The proportion (%) of macrolide resistance gene profiles in *Salmonella enterica* divided by serovars

Serovars/[*]Macrolide	[1]None	[2]*mph*(A)	[3]*mef*(B)	[4]*msr*(E)	[5]*mph*(A), *m*	[6]*mef*(B), *mp*	[7]*mef*(A), *ms*	[8]*mef*(B), *ms*	Grand Total
Agona	98.04%	0.86%	0.86%	0.12%	0.12%	0.00%	0.00%	0.00%	100.00%
Anatum	99.91%	0.09%	0.00%	0.00%	0.00%	0.00%	0.00%	0.00%	100.00%
Braenderup	99.84%	0.16%	0.00%	0.00%	0.00%	0.00%	0.00%	0.00%	100.00%
Derby	98.95%	1.05%	0.00%	0.00%	0.00%	0.00%	0.00%	0.00%	100.00%
Dublin	99.57%	0.29%	0.00%	0.00%	0.00%	0.00%	0.14%	0.00%	100.00%
Enteritidis	99.97%	0.03%	0.00%	0.00%	0.00%	0.00%	0.00%	0.00%	100.00%
Heidelberg	99.84%	0.16%	0.00%	0.00%	0.00%	0.00%	0.00%	0.00%	100.00%
I 1,4,[5],12:i:-	99.71%	0.29%	0.00%	0.00%	0.00%	0.00%	0.00%	0.00%	100.00%
Infantis	99.73%	0.08%	0.19%	0.00%	0.00%	0.00%	0.00%	0.00%	100.00%
Javiana	99.83%	0.17%	0.00%	0.00%	0.00%	0.00%	0.00%	0.00%	100.00%
Kentucky	99.63%	0.23%	0.00%	0.00%	0.09%	0.05%	0.00%	0.00%	100.00%
Mbandaka	100.00%	0.00%	0.00%	0.00%	0.00%	0.00%	0.00%	0.00%	100.00%
Montevideo	99.91%	0.09%	0.00%	0.00%	0.00%	0.00%	0.00%	0.00%	100.00%
Muenchen	99.93%	0.00%	0.07%	0.00%	0.00%	0.00%	0.00%	0.00%	100.00%
Newport	98.68%	1.32%	0.00%	0.00%	0.00%	0.00%	0.00%	0.00%	100.00%
Others	99.54%	0.34%	0.09%	0.02%	0.01%	0.00%	0.00%	0.00%	100.00%
Reading	100.00%	0.00%	0.00%	0.00%	0.00%	0.00%	0.00%	0.00%	100.00%
Saintpaul	99.34%	0.55%	0.11%	0.00%	0.00%	0.00%	0.00%	0.00%	100.00%
Schwarzengrund	99.50%	0.50%	0.00%	0.00%	0.00%	0.00%	0.00%	0.00%	100.00%
Senftenberg	99.77%	0.23%	0.00%	0.00%	0.00%	0.00%	0.00%	0.00%	100.00%
Thompson	99.70%	0.30%	0.00%	0.00%	0.00%	0.00%	0.00%	0.00%	100.00%
Typhimurium	98.69%	1.00%	0.14%	0.14%	0.02%	0.00%	0.00%	0.02%	100.00%
**Grand Total**	**99.49%**	**0.39%**	**0.07%**	**0.03%**	**0.01%**	**0.00%**	**0.00%**	**0.00%**	**100.00%**

Note: The percentage (proportion) of ARGs was calculated by the number of positive-predicted ARGs (each cell) divided by the total number of isolates (each row)
