# Supplementary material for: An Overview of Antimicrobial Resistance Profiles of Publicly Available Salmonella Genomes with Sufficient Quality and Metadata
Source: Foodborne Pathog Dis. 2023 Sep 4;20(9):405–13. doi: 10.1089/fpd.2022.0080 (PMC10510693; doi:10.1089/fpd.2022.0080)
Supplement: Supplemental data [file Suppl_FigureS4.docx]

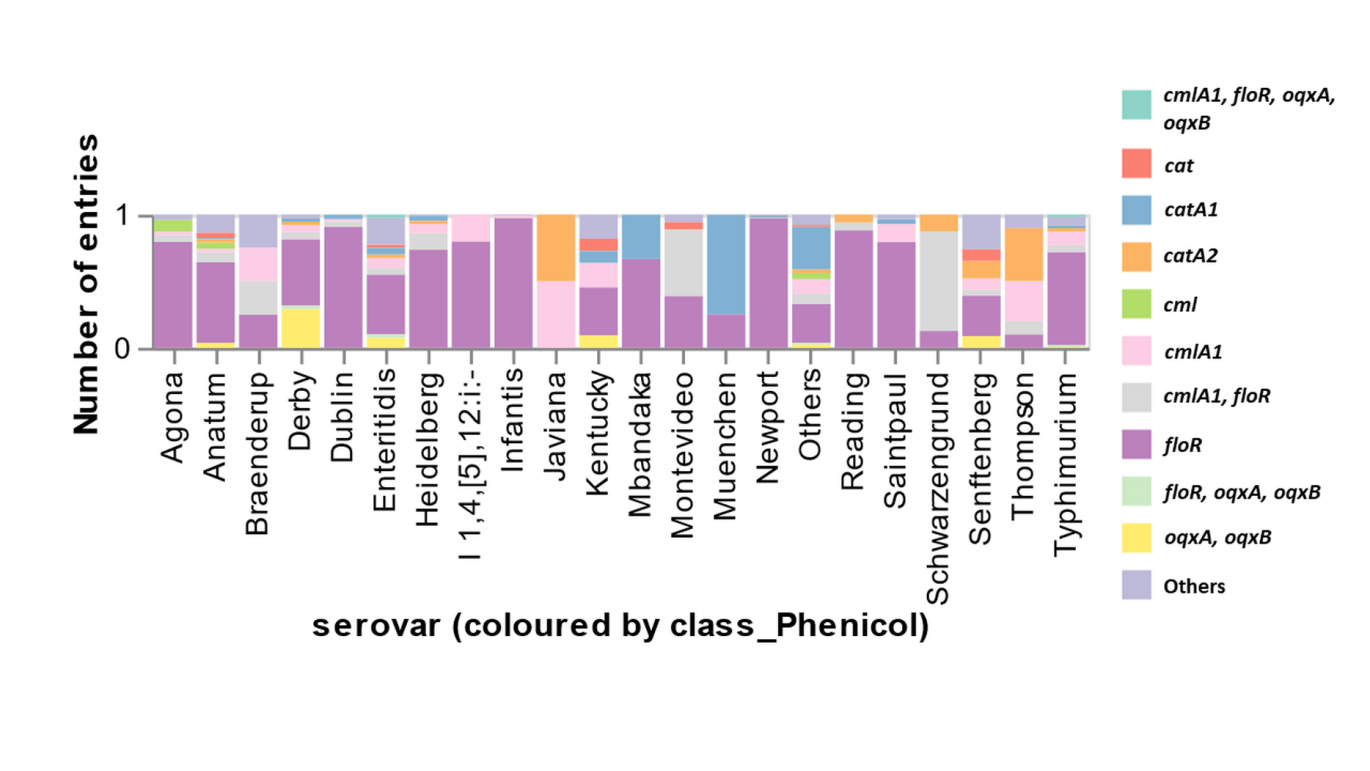


**SUPPLEMENTARY FIG. S4.** The proportion of phenicol resistance gene profiles in *Salmonella enterica* (positive prediction = 3,766 isolates) was categorized by serovar.
