# Supplementary material for: An Overview of Antimicrobial Resistance Profiles of Publicly Available Salmonella Genomes with Sufficient Quality and Metadata
Source: Foodborne Pathog Dis. 2023 Sep 4;20(9):405–13. doi: 10.1089/fpd.2022.0080 (PMC10510693; doi:10.1089/fpd.2022.0080)
Supplement: Supplemental data [file Supp_DataS8.pdf]

SUPPLEMENTARY DATA S8. THE PROPORTION (%) OF PHENICOL RESISTANCE GENE PROFILES IN *SALMONELLA ENTERICA* IN THIS STUDY

The proportion (%) of phenicol resistance gene profiles in *Salmonella enterica* divided by isolation sources

Sources/[*]Phenicol re	[1]None	[2]*floR*	[3]*catA1*	[4]*cmlA1*	[5]*cmlA1, flo*	[6]Others	[7]*catA2*	[8]*oqxA, oqx*	[9]*cml*	[10]*floR, oqxA*	[11]*cmlA1, fl*	[12]cat	Grand Total
Avian	91.45%	7.25%	0.13%	0.42%	0.25%	0.23%	0.07%	0.10%	0.00%	0.03%	0.04%	0.03%	100.00%
Bovine	74.41%	23.55%	0.45%	0.32%	0.88%	0.10%	0.06%	0.03%	0.00%	0.00%	0.00%	0.19%	100.00%
Environmental	94.99%	4.20%	0.07%	0.09%	0.30%	0.09%	0.02%	0.04%	0.19%	0.00%	0.00%	0.02%	100.00%
Feed	94.66%	5.34%	0.00%	0.00%	0.00%	0.00%	0.00%	0.00%	0.00%	0.00%	0.00%	0.00%	100.00%
Food	96.84%	2.00%	0.37%	0.28%	0.09%	0.14%	0.09%	0.14%	0.05%	0.00%	0.00%	0.00%	100.00%
Human	93.81%	3.55%	1.34%	0.28%	0.22%	0.49%	0.08%	0.04%	0.00%	0.13%	0.07%	0.01%	100.00%
Nut/Bean	99.12%	0.71%	0.00%	0.00%	0.18%	0.00%	0.00%	0.00%	0.00%	0.00%	0.00%	0.00%	100.00%
Others	93.81%	4.79%	0.11%	0.29%	0.55%	0.11%	0.22%	0.04%	0.04%	0.00%	0.00%	0.04%	100.00%
Plant	100.00%	0.00%	0.00%	0.00%	0.00%	0.00%	0.00%	0.00%	0.00%	0.00%	0.00%	0.00%	100.00%
Swine	81.57%	10.47%	0.38%	3.25%	1.43%	0.92%	0.80%	0.16%	0.76%	0.06%	0.16%	0.03%	100.00%
Water	98.28%	1.29%	0.00%	0.00%	0.00%	0.14%	0.02%	0.20%	0.00%	0.00%	0.00%	0.07%	100.00%
**Grand Total**	**92.06%**	**5.94%**	**0.51%**	**0.45%**	**0.35%**	**0.30%**	**0.12%**	**0.08%**	**0.08%**	**0.05%**	**0.04%**	**0.03%**	**100.00%**

*	Phenicol resistance gene profiles
1	None;
2	*floR* ;
3	*catA1*;
4	*cmlA1*;
5	*cmlA1, floR*;
6	Others;
7	*catA2*;
8	*oqxA* , *oqxB* ;
9	*cml* ;
10	*floR* , *oqxA* , *oqxB* ;
11	*cmlA1, floR, oqxA, oqxB*;
12	*cat* ;

The proportion (%) of phenicol resistance gene profiles in *Salmonella enterica* divided by serovars

Serovars/[*]Phenicol r	[1]None	[2]*floR*	[3]*catA1*	[4]*cmlA1*	[5]*cmlA1, flo*	[6]Others	[7]*catA2*	[8]*oqxA, oqx*	[9]*cml*	[10]*floR, oqxA*	[11]*cmlA1, fl*	[12]cat	Grand Total
Agona	90.46%	7.58%	0.00%	0.24%	0.49%	0.37%	0.00%	0.00%	0.86%	0.00%	0.00%	0.00%	100.00%
Anatum	92.72%	4.40%	0.09%	0.18%	0.54%	0.99%	0.18%	0.27%	0.36%	0.00%	0.00%	0.27%	100.00%
Braenderup	99.36%	0.16%	0.00%	0.16%	0.16%	0.16%	0.00%	0.00%	0.00%	0.00%	0.00%	0.00%	100.00%
Derby	93.37%	3.32%	0.17%	0.35%	0.35%	0.17%	0.17%	1.92%	0.00%	0.17%	0.00%	0.00%	100.00%
Dublin	28.22%	65.12%	2.46%	1.01%	3.18%	0.00%	0.00%	0.00%	0.00%	0.00%	0.00%	0.00%	100.00%
Enteritidis	99.39%	0.27%	0.03%	0.05%	0.03%	0.12%	0.02%	0.05%	0.00%	0.02%	0.02%	0.02%	100.00%
Heidelberg	89.77%	7.53%	0.40%	0.71%	1.27%	0.08%	0.24%	0.00%	0.00%	0.00%	0.00%	0.00%	100.00%
I 1,4,[5],12:i:-	98.53%	1.18%	0.00%	0.29%	0.00%	0.00%	0.00%	0.00%	0.00%	0.00%	0.00%	0.00%	100.00%
Infantis	63.40%	35.64%	0.04%	0.77%	0.00%	0.15%	0.00%	0.00%	0.00%	0.00%	0.00%	0.00%	100.00%
Javiana	99.83%	0.00%	0.00%	0.09%	0.00%	0.00%	0.09%	0.00%	0.00%	0.00%	0.00%	0.00%	100.00%
Kentucky	99.49%	0.19%	0.05%	0.09%	0.00%	0.09%	0.00%	0.05%	0.00%	0.00%	0.00%	0.05%	100.00%
Mbandaka	99.55%	0.30%	0.15%	0.00%	0.00%	0.00%	0.00%	0.00%	0.00%	0.00%	0.00%	0.00%	100.00%
Montevideo	98.41%	0.62%	0.00%	0.00%	0.80%	0.09%	0.00%	0.00%	0.00%	0.00%	0.00%	0.09%	100.00%
Muenchen	99.45%	0.14%	0.42%	0.00%	0.00%	0.00%	0.00%	0.00%	0.00%	0.00%	0.00%	0.00%	100.00%
Newport	87.53%	12.14%	0.22%	0.00%	0.04%	0.04%	0.00%	0.00%	0.00%	0.00%	0.00%	0.04%	100.00%
Others	95.96%	1.18%	1.29%	0.44%	0.32%	0.32%	0.11%	0.10%	0.19%	0.04%	0.00%	0.05%	100.00%
Reading	96.94%	2.70%	0.00%	0.00%	0.18%	0.00%	0.18%	0.00%	0.00%	0.00%	0.00%	0.00%	100.00%
Saintpaul	96.83%	2.51%	0.11%	0.44%	0.00%	0.11%	0.00%	0.00%	0.00%	0.00%	0.00%	0.00%	100.00%
Schwarzengrund	98.66%	0.17%	0.00%	0.00%	1.01%	0.00%	0.17%	0.00%	0.00%	0.00%	0.00%	0.00%	100.00%
Senftenberg	97.35%	0.81%	0.00%	0.23%	0.12%	0.69%	0.35%	0.23%	0.00%	0.00%	0.00%	0.23%	100.00%
Thompson	98.51%	0.15%	0.00%	0.45%	0.15%	0.15%	0.60%	0.00%	0.00%	0.00%	0.00%	0.00%	100.00%
Typhimurium	84.19%	11.03%	0.32%	1.63%	0.84%	0.98%	0.39%	0.07%	0.00%	0.26%	0.30%	0.00%	100.00%
**Grand Total**	**92.06%**	**5.94%**	**0.51%**	**0.45%**	**0.35%**	**0.30%**	**0.12%**	**0.08%**	**0.08%**	**0.05%**	**0.04%**	**0.03%**	**100.00%**

Note: The percentage (proportion) of ARGs was calculated by the number of positive-predicted ARGs (each cell) divided by the total number of isolates (each row)
