# Supplementary material for: An Overview of Antimicrobial Resistance Profiles of Publicly Available Salmonella Genomes with Sufficient Quality and Metadata
Source: Foodborne Pathog Dis. 2023 Sep 4;20(9):405–13. doi: 10.1089/fpd.2022.0080 (PMC10510693; doi:10.1089/fpd.2022.0080)
Supplement: Supplemental data [file Supp_DataS9.pdf]

SUPPLEMENTARY DATA S9. THE PROPORTION (%) OF POLYMYXIN RESISTANCE GENE PROFILES IN *SALMONELLA ENTERICA* IN THIS STUDY

The proportion (%) of polymyxin resistance gene profiles in *Salmonella enterica* divided by isolation source

Sources/[*]Polymyxin	[1]None	[2]*mcr-9*	[3]*mcr-1.1*	[4]*mcr-5.1*	[5]*mcr-3.1*	[6]*mcr-1.1, m*	[7]*mcr-1.1, m*	[8]*mcr-1.26*	[9]*mcr-4.6*	[10]*mcr-3.20*	[11]*mcr-3.21*	[12]*mcr-1.2*	[13]*mcr-4.2*	Grand Total
Avian	96.86%	2.86%	0.17%	0.10%	0.00%	0.00%	0.00%	0.00%	0.00%	0.00%	0.00%	0.01%	0.00%	100.00%
Bovine	99.55%	0.39%	0.00%	0.03%	0.00%	0.00%	0.00%	0.00%	0.00%	0.00%	0.00%	0.00%	0.03%	100.00%
Environmental	99.59%	0.34%	0.00%	0.07%	0.00%	0.00%	0.00%	0.00%	0.00%	0.00%	0.00%	0.00%	0.00%	100.00%
Feed	99.14%	0.86%	0.00%	0.00%	0.00%	0.00%	0.00%	0.00%	0.00%	0.00%	0.00%	0.00%	0.00%	100.00%
Food	98.19%	1.76%	0.05%	0.00%	0.00%	0.00%	0.00%	0.00%	0.00%	0.00%	0.00%	0.00%	0.00%	100.00%
Human	99.65%	0.23%	0.07%	0.00%	0.00%	0.04%	0.01%	0.01%	0.00%	0.00%	0.00%	0.00%	0.00%	100.00%
Nut/Bean	99.82%	0.18%	0.00%	0.00%	0.00%	0.00%	0.00%	0.00%	0.00%	0.00%	0.00%	0.00%	0.00%	100.00%
Others	99.45%	0.29%	0.26%	0.00%	0.00%	0.00%	0.00%	0.00%	0.00%	0.00%	0.00%	0.00%	0.00%	100.00%
Plant	100.00%	0.00%	0.00%	0.00%	0.00%	0.00%	0.00%	0.00%	0.00%	0.00%	0.00%	0.00%	0.00%	100.00%
Swine	97.55%	1.56%	0.29%	0.06%	0.29%	0.00%	0.10%	0.00%	0.06%	0.06%	0.03%	0.00%	0.00%	100.00%
Water	99.98%	0.02%	0.00%	0.00%	0.00%	0.00%	0.00%	0.00%	0.00%	0.00%	0.00%	0.00%	0.00%	100.00%
**Grand Total**	**98.82%**	**0.99%**	**0.09%**	**0.04%**	**0.02%**	**0.01%**	**0.01%**	**0.00%**	**0.00%**	**0.00%**	**0.00%**	**0.00%**	**0.00%**	**100.00%**

*	Polymyxin resistance gene profiles
1	None;
2	*mcr-9*;
3	*mcr-1.1*;
4	*mcr-5.1*;
5	*mcr-3.1*;
6	*mcr-1.1*, *mcr-1.2*, *mcr-1.3*, *mcr-1.8*, *mcr-1.11*, *mcr-1.12*, *mcr-1.14*, *mcr-1.26*, *mcr-1.27*;
7	*mcr-1.1*, *mcr-9*;
8	*mcr-1.26*;
9	*mcr-4.6*;
10	*mcr-3.20*;
11	*mcr-3.21*;
12	*mcr-1.2*;
13	*mcr-4.2*;

The proportion (%) of polymyxin resistance gene profiles in *Salmonella enterica* divided by serovars

Serovars/[*]Polymyxir	[1]None	[2]*mcr-9*	[3]*mcr-1.1*	[4]*mcr-5.1*	[5]*mcr-3.1*	[6]*mcr-1.1, m*	[7]*mcr-1.1, m*	[8]*mcr-1.26*	[9]*mcr-4.6*	[10]*mcr-3.20*	[11]*mcr-3.21*	[12]*mcr-1.2*	[13]*mcr-4.2*	Grand Total
Agona	98.66%	0.86%	0.49%	0.00%	0.00%	0.00%	0.00%	0.00%	0.00%	0.00%	0.00%	0.00%	0.00%	100.00%
Anatum	99.01%	0.45%	0.36%	0.00%	0.00%	0.00%	0.18%	0.00%	0.00%	0.00%	0.00%	0.00%	0.00%	100.00%
Braenderup	99.68%	0.32%	0.00%	0.00%	0.00%	0.00%	0.00%	0.00%	0.00%	0.00%	0.00%	0.00%	0.00%	100.00%
Derby	100.00%	0.00%	0.00%	0.00%	0.00%	0.00%	0.00%	0.00%	0.00%	0.00%	0.00%	0.00%	0.00%	100.00%
Dublin	100.00%	0.00%	0.00%	0.00%	0.00%	0.00%	0.00%	0.00%	0.00%	0.00%	0.00%	0.00%	0.00%	100.00%
Enteritidis	99.97%	0.00%	0.02%	0.00%	0.00%	0.00%	0.00%	0.02%	0.00%	0.00%	0.00%	0.00%	0.00%	100.00%
Heidelberg	89.93%	10.07%	0.00%	0.00%	0.00%	0.00%	0.00%	0.00%	0.00%	0.00%	0.00%	0.00%	0.00%	100.00%
I 1,4,[5],12:i:-	99.85%	0.00%	0.00%	0.15%	0.00%	0.00%	0.00%	0.00%	0.00%	0.00%	0.00%	0.00%	0.00%	100.00%
Infantis	99.92%	0.04%	0.04%	0.00%	0.00%	0.00%	0.00%	0.00%	0.00%	0.00%	0.00%	0.00%	0.00%	100.00%
Javiana	99.91%	0.09%	0.00%	0.00%	0.00%	0.00%	0.00%	0.00%	0.00%	0.00%	0.00%	0.00%	0.00%	100.00%
Kentucky	99.95%	0.05%	0.00%	0.00%	0.00%	0.00%	0.00%	0.00%	0.00%	0.00%	0.00%	0.00%	0.00%	100.00%
Mbandaka	98.96%	1.04%	0.00%	0.00%	0.00%	0.00%	0.00%	0.00%	0.00%	0.00%	0.00%	0.00%	0.00%	100.00%
Montevideo	99.91%	0.09%	0.00%	0.00%	0.00%	0.00%	0.00%	0.00%	0.00%	0.00%	0.00%	0.00%	0.00%	100.00%
Muenchen	99.93%	0.07%	0.00%	0.00%	0.00%	0.00%	0.00%	0.00%	0.00%	0.00%	0.00%	0.00%	0.00%	100.00%
Newport	99.93%	0.00%	0.07%	0.00%	0.00%	0.00%	0.00%	0.00%	0.00%	0.00%	0.00%	0.00%	0.00%	100.00%
Others	98.71%	0.96%	0.14%	0.11%	0.01%	0.04%	0.00%	0.00%	0.01%	0.00%	0.00%	0.01%	0.00%	100.00%
Reading	99.82%	0.18%	0.00%	0.00%	0.00%	0.00%	0.00%	0.00%	0.00%	0.00%	0.00%	0.00%	0.00%	100.00%
Saintpaul	89.41%	10.59%	0.00%	0.00%	0.00%	0.00%	0.00%	0.00%	0.00%	0.00%	0.00%	0.00%	0.00%	100.00%
Schwarzengrund	98.32%	1.68%	0.00%	0.00%	0.00%	0.00%	0.00%	0.00%	0.00%	0.00%	0.00%	0.00%	0.00%	100.00%
Senftenberg	98.27%	1.73%	0.00%	0.00%	0.00%	0.00%	0.00%	0.00%	0.00%	0.00%	0.00%	0.00%	0.00%	100.00%
Thompson	99.26%	0.74%	0.00%	0.00%	0.00%	0.00%	0.00%	0.00%	0.00%	0.00%	0.00%	0.00%	0.00%	100.00%
Typhimurium	98.55%	0.96%	0.21%	0.04%	0.12%	0.00%	0.04%	0.02%	0.00%	0.04%	0.02%	0.00%	0.02%	100.00%
**Grand Total**	**98.82%**	**0.99%**	**0.09%**	**0.04%**	**0.02%**	**0.01%**	**0.01%**	**0.00%**	**0.00%**	**0.00%**	**0.00%**	**0.00%**	**0.00%**	**100.00%**

Note: The percentage (proportion) of ARGs was calculated by the number of positive-predicted ARGs (each cell) divided by the total number of isolates (each row)
