# Supplementary material for: An Overview of Antimicrobial Resistance Profiles of Publicly Available Salmonella Genomes with Sufficient Quality and Metadata
Source: Foodborne Pathog Dis. 2023 Sep 4;20(9):405–13. doi: 10.1089/fpd.2022.0080 (PMC10510693; doi:10.1089/fpd.2022.0080)
Supplement: Supplemental data [file Supp_DataS10.pdf]

SUPPLEMENTARY TABLE S10. THE PROPORTION (%) OF TETRACYCLINE RESISTANCE GENE PROFILES IN *SALMONELLA ENTERICA* IN THIS STUDY

The proportion (%) of tetracycline resistance gene profiles in *Salmonella enterica* divided by isolation sources

**Sources/[*]Tetracyclin**	**[1]None**	**[2]*tet*(A)**	**[3]*tet*(B)**	**[4]*tet*(G)**	**[5]*tet*(C)**	**[6]Others**	**[7]*tet*(A), *tet*(**	**[8]*tet*(A), *tet*(**	**[9]*tet*(A), *tet*(**	**[10]*tet*(A), *tet***	**[11]*tet*(D)**	**[12]*tet*(B), *tet***	**Grand Total**
Avian	56.12%	26.10%	15.90%	0.21%	0.61%	0.11%	0.12%	0.34%	0.00%	0.39%	0.08%	0.01%	100.00%
Bovine	63.36%	25.56%	3.93%	2.44%	2.18%	0.68%	0.42%	0.36%	0.75%	0.10%	0.10%	0.13%	100.00%
Environmental	86.84%	7.96%	3.67%	0.37%	0.44%	0.16%	0.23%	0.12%	0.18%	0.02%	0.02%	0.00%	100.00%
Feed	82.79%	12.22%	2.41%	1.55%	0.86%	0.17%	0.00%	0.00%	0.00%	0.00%	0.00%	0.00%	100.00%
Food	86.86%	9.19%	2.97%	0.37%	0.23%	0.23%	0.05%	0.00%	0.09%	0.00%	0.00%	0.00%	100.00%
Human	84.31%	7.36%	6.21%	0.93%	0.12%	0.27%	0.27%	0.15%	0.17%	0.01%	0.09%	0.11%	100.00%
Nut/Bean	98.06%	1.77%	0.00%	0.00%	0.00%	0.00%	0.18%	0.00%	0.00%	0.00%	0.00%	0.00%	100.00%
Others	89.01%	5.64%	2.54%	1.25%	0.52%	0.29%	0.41%	0.07%	0.04%	0.04%	0.18%	0.00%	100.00%
Plant	98.19%	0.15%	1.21%	0.00%	0.45%	0.00%	0.00%	0.00%	0.00%	0.00%	0.00%	0.00%	100.00%
Swine	46.44%	16.74%	23.04%	3.66%	4.30%	2.29%	1.59%	1.15%	0.00%	0.00%	0.38%	0.41%	100.00%
Water	95.32%	3.34%	0.72%	0.16%	0.34%	0.11%	0.00%	0.00%	0.00%	0.00%	0.00%	0.00%	100.00%
**Grand Total**	**76.15%**	**12.95%**	**8.00%**	**0.88%**	**0.74%**	**0.36%**	**0.29%**	**0.24%**	**0.13%**	**0.10%**	**0.09%**	**0.07%**	**100.00%**

*	**Tetracycline resistance gene profiles**
1	None;
2	*tet* (A);
3	*tet* (B);
4	*tet* (G);
5	*tet* (C);
6	Others;
7	*tet* (A), *tet* (M);
8	*tet* (A), *tet(* B);
9	*tet* (A), *tet* (B), *tet(* O);
10	*tet* (A), *tet* (C);
11	*tet* (D);
12	*tet* (B), *tet* (M);

The proportion (%) of tetracycline resistance gene profiles in *Salmonella enterica* divided by serovars

**Serovars/[*]Tetracycli**	**[1]None**	**[2]*tet*(A)**	**[3]*tet*(B)**	**[4]*tet*(G)**	**[5]*tet*(C)**	**[6]Others**	**[7]*tet*(A), *tet*(**	**[8]*tet*(A), *tet*(**	**[9]*tet*(A), *tet*(**	**[10]*tet*(A), *tet***	**[11]*tet*(D)**	**[12]*tet*(B), *tet***	**Grand Total**
Agona	72.98%	16.87%	7.21%	0.37%	0.24%	0.12%	1.22%	0.49%	0.00%	0.00%	0.49%	0.00%	100.00%
Anatum	73.94%	7.01%	4.76%	0.00%	12.76%	0.90%	0.18%	0.09%	0.00%	0.09%	0.27%	0.00%	100.00%
Braenderup	96.82%	2.54%	0.16%	0.16%	0.16%	0.00%	0.00%	0.00%	0.00%	0.00%	0.16%	0.00%	100.00%
Derby	30.19%	50.96%	16.93%	0.17%	0.52%	0.17%	0.52%	0.35%	0.00%	0.00%	0.17%	0.00%	100.00%
Dublin	23.30%	74.53%	2.03%	0.00%	0.00%	0.14%	0.00%	0.00%	0.00%	0.00%	0.00%	0.00%	100.00%
Enteritidis	95.66%	3.91%	0.38%	0.00%	0.00%	0.03%	0.02%	0.00%	0.00%	0.00%	0.00%	0.00%	100.00%
Heidelberg	73.28%	6.11%	8.64%	0.00%	3.49%	1.03%	0.16%	2.22%	4.76%	0.32%	0.00%	0.00%	100.00%
I 1,4,[5],12:i:-	89.53%	4.87%	4.87%	0.44%	0.00%	0.15%	0.00%	0.00%	0.00%	0.00%	0.15%	0.00%	100.00%
Infantis	42.00%	57.24%	0.50%	0.04%	0.08%	0.04%	0.00%	0.00%	0.00%	0.00%	0.11%	0.00%	100.00%
Javiana	99.65%	0.26%	0.09%	0.00%	0.00%	0.00%	0.00%	0.00%	0.00%	0.00%	0.00%	0.00%	100.00%
Kentucky	30.86%	7.78%	60.52%	0.00%	0.46%	0.09%	0.05%	0.19%	0.00%	0.05%	0.00%	0.00%	100.00%
Mbandaka	89.70%	4.48%	5.67%	0.00%	0.15%	0.00%	0.00%	0.00%	0.00%	0.00%	0.00%	0.00%	100.00%
Montevideo	94.43%	1.50%	1.59%	0.00%	1.41%	0.18%	0.80%	0.00%	0.00%	0.09%	0.00%	0.00%	100.00%
Muenchen	86.48%	13.38%	0.00%	0.00%	0.14%	0.00%	0.00%	0.00%	0.00%	0.00%	0.00%	0.00%	100.00%
Newport	85.95%	13.36%	0.40%	0.04%	0.07%	0.07%	0.00%	0.11%	0.00%	0.00%	0.00%	0.00%	100.00%
Others	88.51%	7.35%	2.60%	0.16%	0.58%	0.16%	0.39%	0.11%	0.00%	0.01%	0.11%	0.01%	100.00%
Reading	67.45%	26.98%	0.18%	0.00%	4.32%	0.36%	0.18%	0.18%	0.00%	0.36%	0.00%	0.00%	100.00%
Saintpaul	65.61%	18.23%	14.63%	0.00%	0.11%	0.00%	0.87%	0.55%	0.00%	0.00%	0.00%	0.00%	100.00%
Schwarzengrund	81.71%	12.42%	4.53%	0.00%	1.01%	0.00%	0.00%	0.17%	0.00%	0.00%	0.17%	0.00%	100.00%
Senftenberg	93.54%	4.73%	0.69%	0.00%	0.00%	0.69%	0.12%	0.00%	0.00%	0.00%	0.23%	0.00%	100.00%
Thompson	98.07%	0.89%	0.00%	0.00%	0.00%	0.00%	0.00%	0.00%	0.00%	0.00%	1.04%	0.00%	100.00%
Typhimurium	44.54%	17.66%	26.03%	6.78%	0.23%	1.82%	0.82%	0.84%	0.00%	0.65%	0.07%	0.56%	100.00%
**Grand Total**	**76.15%**	**12.95%**	**8.00%**	**0.88%**	**0.74%**	**0.36%**	**0.29%**	**0.24%**	**0.13%**	**0.10%**	**0.09%**	**0.07%**	**100.00%**

Note: The percentage (proportion) of ARGs was calculated by the number of positive-predicted ARGs (each cell) divided by the total number of isolates (each row)
