# Supplementary material for: An Overview of Antimicrobial Resistance Profiles of Publicly Available Salmonella Genomes with Sufficient Quality and Metadata
Source: Foodborne Pathog Dis. 2023 Sep 4;20(9):405–13. doi: 10.1089/fpd.2022.0080 (PMC10510693; doi:10.1089/fpd.2022.0080)
Supplement: Supplemental data [file Suppl_Data.pdf]

## SUPPLEMENTARY DATA S1. THE PERCENTAGE OF *SALMONELLA ENTERICA* DISTRIBUTION IN THIS STUDY

**The percentage of *Salmonella enterica* distribution divided by serovars and isolation sources**

| Serovars/Sources | Human | Avian | Environmen | Water | Swine | Bovine | Others | Food | Plant | Feed | Nut/Bean | Grand Total |
|---|---|---|---|---|---|---|---|---|---|---|---|---|
| Agona | 0.38% | 0.33% | 0.35% | 0.04% | 0.18% | 0.15% | 0.09% | 0.10% | 0.01% | 0.07% | 0.03% | 1.72% |
| Anatum | 0.23% | 0.27% | 0.24% | 0.19% | 0.59% | 0.40% | 0.18% | 0.11% | 0.02% | 0.04% | 0.07% | 2.35% |
| Braenderup | 0.38% | 0.29% | 0.18% | 0.16% | 0.01% | 0.03% | 0.20% | 0.04% | 0.02% | 0.01% | 0.02% | 1.33% |
| Derby | 0.07% | 0.22% | 0.08% | 0.00% | 0.66% | 0.03% | 0.03% | 0.07% | 0.00% | 0.03% | 0.01% | 1.21% |
| Dublin | 0.25% | 0.01% | 0.07% | 0.01% | 0.01% | 1.03% | 0.03% | 0.03% | 0.00% | 0.01% | 0.00% | 1.46% |
| Enteritidis | 8.54% | 3.16% | 1.37% | 0.25% | 0.02% | 0.05% | 0.09% | 0.24% | 0.00% | 0.04% | 0.08% | 13.84% |
| Heidelberg | 0.47% | 1.57% | 0.20% | 0.04% | 0.08% | 0.09% | 0.03% | 0.16% | 0.00% | 0.01% | 0.01% | 2.66% |
| I 1,4,[5],12:i:- | 0.59% | 0.34% | 0.07% | 0.19% | 0.04% | 0.05% | 0.04% | 0.03% | 0.00% | 0.05% | 0.04% | 1.43% |
| Infantis | 0.79% | 2.76% | 0.66% | 0.34% | 0.42% | 0.13% | 0.11% | 0.12% | 0.03% | 0.13% | 0.01% | 5.50% |
| Javiana | 1.09% | 0.06% | 0.31% | 0.43% | 0.00% | 0.00% | 0.29% | 0.07% | 0.14% | 0.00% | 0.00% | 2.41% |
| Kentucky | 0.22% | 3.70% | 0.18% | 0.06% | 0.02% | 0.12% | 0.06% | 0.15% | 0.00% | 0.03% | 0.00% | 4.55% |
| Mbandaka | 0.11% | 0.21% | 0.52% | 0.07% | 0.07% | 0.17% | 0.05% | 0.16% | 0.00% | 0.04% | 0.02% | 1.41% |
| Montevideo | 0.29% | 0.16% | 0.47% | 0.35% | 0.03% | 0.68% | 0.13% | 0.14% | 0.03% | 0.05% | 0.04% | 2.38% |
| Muenchen | 0.41% | 0.93% | 0.35% | 0.45% | 0.10% | 0.26% | 0.19% | 0.21% | 0.03% | 0.01% | 0.09% | 3.04% |
| Newport | 2.00% | 0.29% | 0.96% | 1.09% | 0.11% | 0.47% | 0.48% | 0.19% | 0.06% | 0.02% | 0.05% | 5.73% |
| Others | 7.57% | 3.07% | 3.89% | 4.16% | 2.11% | 1.81% | 2.97% | 1.94% | 0.92% | 0.51% | 0.38% | 29.35% |
| Reading | 0.13% | 0.79% | 0.08% | 0.01% | 0.03% | 0.06% | 0.05% | 0.03% | 0.00% | 0.00% | 0.00% | 1.17% |
| Saintpaul | 0.52% | 0.66% | 0.19% | 0.28% | 0.07% | 0.02% | 0.06% | 0.09% | 0.00% | 0.01% | 0.03% | 1.93% |
| Schwarzengrund | 0.09% | 0.75% | 0.15% | 0.02% | 0.04% | 0.04% | 0.03% | 0.09% | 0.01% | 0.04% | 0.00% | 1.26% |
| Senftenberg | 0.10% | 0.39% | 0.53% | 0.05% | 0.08% | 0.08% | 0.06% | 0.27% | 0.01% | 0.05% | 0.21% | 1.83% |
| Thompson | 0.36% | 0.22% | 0.12% | 0.36% | 0.00% | 0.04% | 0.09% | 0.06% | 0.08% | 0.01% | 0.06% | 1.42% |
| Typhimurium | 4.50% | 2.29% | 0.92% | 0.78% | 1.93% | 0.76% | 0.47% | 0.26% | 0.02% | 0.06% | 0.05% | 12.04% |
| **Grand Total** | **29.10%** | **22.50%** | **11.89%** | **9.33%** | **6.62%** | **6.49%** | **5.72%** | **4.54%** | **1.40%** | **1.22%** | **1.19%** | **100.00%** |

**Note**: The percentage was calculated by the number of isolates in each cell divided by the total number of samples (47,452).

## SUPPLEMENTARY DATA S2. THE PERCENTAGE OF POSITIVE-PREDICTED AMR IN *SALMONELLA ENTERICA* IN THIS STUDY

**The percentage of positive-predicted AMR in *Salmonella enterica* divided by isolation sources and antimicrobial classes**

| **Sources/[*]Antimicrob** | **[1]Aminoglyc** | **[2]β-lactam** | **[3]Fluoroquir** | **[4]Folate patl** | **[5]Macrolide** | **[6]Phenicol** | **[7]Polymyxin** | **[8]Tetracycline** |
|---|---|---|---|---|---|---|---|---|
| Human | 28.41% | 4.77% | 1.09% | 4.79% | 0.19% | 1.80% | 0.10% | 4.56% |
| Avian | 22.28% | 4.68% | 0.81% | 6.12% | 0.08% | 1.92% | 0.71% | 9.87% |
| Environmental | 11.78% | 0.97% | 0.24% | 1.25% | 0.03% | 0.60% | 0.05% | 1.57% |
| Water | 9.10% | 0.20% | 0.08% | 0.35% | 0.00% | 0.16% | 0.00% | 0.44% |
| Swine | 6.58% | 2.44% | 0.64% | 2.87% | 0.11% | 1.22% | 0.16% | 3.55% |
| Bovine | 6.44% | 1.80% | 0.17% | 2.09% | 0.04% | 1.66% | 0.03% | 2.38% |
| Food | 4.49% | 0.31% | 0.16% | 0.45% | 0.01% | 0.14% | 0.08% | 0.60% |
| Plant | 1.39% | 0.01% | 0.01% | 0.01% | 0.00% | 0.00% | 0.00% | 0.03% |
| Feed | 1.22% | 0.14% | 0.02% | 0.17% | 0.01% | 0.07% | 0.01% | 0.21% |
| Nut/Bean | 1.18% | 0.02% | 0.00% | 0.02% | 0.00% | 0.01% | 0.00% | 0.02% |
| Others | 5.52% | 0.46% | 0.13% | 0.52% | 0.04% | 0.35% | 0.03% | 0.63% |
| **Grand Total** | **98.39%** | **15.78%** | **3.36%** | **18.63%** | **0.51%** | **7.94%** | **1.18%** | **23.85%** |

| * | **Antimicrobial classes** |
|---|---|
| 1 | Aminoglycoside |
| 2 | β-lactam |
| 3 | Fluoroquinolone |
| 4 | Folate pathway antagonist |
| 5 | Macrolide |
| 6 | Phenicol |
| 7 | Polymyxin |
| 8 | Tetracycline |

**Note**: The percentage of AMR was calculated by the number of positive-predicted AMR in each cell divided by the total number of samples (47,452).

**SUPPLEMENTARY DATA S3. THE PROPORTION (%) OF AMINOGLYCOSIDE RESISTANCE GENE PROFILES IN *SALMONELLA ENTERICA* IN THIS STUDY**

**The proportion (%) of aminoglycoside resistance gene profiles in *Salmonella enterica* divided by isolation sources**

| **Sources/[*]Aminoglyc** | [1]***aac(6')-Iac*** | [2]***aac(3)-IV,*** | [3]**None** | [4]***aac(3)-VIa*** | [5]***aac(3)-IId,*** | [6]**Others** | [7]***aac(6')-Iac*** | [8]***aac(6')-Iac*** | [9]***aac(3)-Id, (*** | [10]***aac(3)-VI(*** | [11]***aac(6')-Ia*** | [12]***aac(6')-Ia*** | **Grand Total** |
|---|---|---|---|---|---|---|---|---|---|---|---|---|---|
| Avian | 82.80% | 7.85% | 1.00% | 3.32% | 1.55% | 0.62% | 0.99% | 0.30% | 0.44% | 0.59% | 0.53% | 0.00% | 100.00% |
| Bovine | 96.69% | 0.16% | 0.78% | 0.65% | 0.16% | 0.32% | 0.00% | 0.94% | 0.16% | 0.06% | 0.03% | 0.03% | 100.00% |
| Environmental | 96.55% | 1.51% | 0.94% | 0.32% | 0.25% | 0.23% | 0.02% | 0.12% | 0.00% | 0.04% | 0.04% | 0.00% | 100.00% |
| Feed | 94.15% | 1.03% | 0.34% | 0.34% | 3.61% | 0.17% | 0.00% | 0.00% | 0.00% | 0.34% | 0.00% | 0.00% | 100.00% |
| Food | 93.69% | 0.51% | 1.07% | 1.02% | 0.65% | 0.65% | 0.84% | 0.14% | 0.51% | 0.46% | 0.46% | 0.00% | 100.00% |
| Human | 94.68% | 0.73% | 2.37% | 0.44% | 0.68% | 0.57% | 0.01% | 0.12% | 0.29% | 0.01% | 0.00% | 0.08% | 100.00% |
| Nut/Bean | 98.76% | 0.00% | 0.88% | 0.00% | 0.35% | 0.00% | 0.00% | 0.00% | 0.00% | 0.00% | 0.00% | 0.00% | 100.00% |
| Others | 94.36% | 0.37% | 3.50% | 0.26% | 0.48% | 0.63% | 0.00% | 0.18% | 0.04% | 0.00% | 0.00% | 0.18% | 100.00% |
| Plant | 99.24% | 0.00% | 0.45% | 0.30% | 0.00% | 0.00% | 0.00% | 0.00% | 0.00% | 0.00% | 0.00% | 0.00% | 100.00% |
| Swine | 89.78% | 3.31% | 0.60% | 1.18% | 1.50% | 2.04% | 0.00% | 0.80% | 0.16% | 0.03% | 0.06% | 0.54% | 100.00% |
| Water | 96.18% | 1.08% | 2.42% | 0.09% | 0.07% | 0.16% | 0.00% | 0.00% | 0.00% | 0.00% | 0.00% | 0.00% | 100.00% |
| **Grand Total** | **92.22%** | **2.55%** | **1.61%** | **1.11%** | **0.80%** | **0.57%** | **0.27%** | **0.25%** | **0.23%** | **0.17%** | **0.15%** | **0.07%** | **100.00%** |

| * | **Aminoglycoside resistance gene profiles** |
|---|---|
| 1 | *aac(6')-Iaa*; |
| 2 | *aac(3)-IV*, *aac(6')-Iaa*; |
| 3 | None; |
| 4 | *aac(3)-VIa*, *aac(6')-Iaa*; |
| 5 | *aac(3)-IId*, *aac(6')-Iaa*; |
| 6 | Others; |
| 7 | *aac(6')-Iaa*, *aac(6')-Ib3*, *aac(6')-Ib-cr*, *grdA*; |
| 8 | *aac(6')-Iaa*, *ant(2'')-Ia*; |
| 9 | *aac(3)-Id*, *aac(6')-Iaa*; |
| 10 | *aac(3)-VIa*, *aac(6')-Iaa*, *aac(6')-Ib3*, *aac(6')-Ib-cr*, *grdA*; |
| 11 | *aac(6')-Iaa*, *grdA*; |
| 12 | *aac(6')-Iaa*, *aac(6')-IIc*; |

**The proportion (%) of aminoglycoside resistance gene profiles in *Salmonella enterica* divided by serovars**

| **Serovars/[*]Aminogly(** | [1]***aac(6')-Iac*** | [2]***aac(3)-IV,*** | [3]**None** | [4]***aac(3)-VIa*** | [5]***aac(3)-IId,*** | [6]**Others** | [7]***aac(6')-Iac*** | [8]***aac(6')-Iac*** | [9]***aac(3)-Id, (*** | [10]***aac(3)-VI(*** | [11]***aac(6')-Ia*** | [12]***aac(6')-Ia*** | **Grand Total** |
|---|---|---|---|---|---|---|---|---|---|---|---|---|---|
| Agona | 93.03% | 1.22% | 0.49% | 1.47% | 1.22% | 2.32% | 0.00% | 0.24% | 0.00% | 0.00% | 0.00% | 0.00% | 100.00% |
| Anatum | 96.95% | 1.26% | 0.63% | 0.27% | 0.18% | 0.54% | 0.00% | 0.00% | 0.00% | 0.00% | 0.00% | 0.18% | 100.00% |
| Braenderup | 96.82% | 0.00% | 0.95% | 1.75% | 0.00% | 0.32% | 0.00% | 0.16% | 0.00% | 0.00% | 0.00% | 0.00% | 100.00% |
| Derby | 93.19% | 1.40% | 0.00% | 4.71% | 0.35% | 0.35% | 0.00% | 0.00% | 0.00% | 0.00% | 0.00% | 0.00% | 100.00% |
| Dublin | 94.36% | 0.00% | 1.30% | 0.00% | 0.29% | 0.00% | 0.00% | 4.05% | 0.00% | 0.00% | 0.00% | 0.00% | 100.00% |
| Enteritidis | 97.35% | 0.08% | 2.10% | 0.15% | 0.15% | 0.17% | 0.00% | 0.00% | 0.00% | 0.00% | 0.00% | 0.00% | 100.00% |
| Heidelberg | 69.55% | 0.56% | 1.27% | 12.61% | 2.46% | 3.01% | 2.22% | 2.22% | 0.00% | 5.63% | 0.32% | 0.16% | 100.00% |
| I 1,4,[5],12:i:- | 93.81% | 0.29% | 1.33% | 1.47% | 3.10% | 0.00% | 0.00% | 0.00% | 0.00% | 0.00% | 0.00% | 0.00% | 100.00% |
| Infantis | 60.41% | 38.17% | 0.46% | 0.57% | 0.00% | 0.23% | 0.00% | 0.15% | 0.00% | 0.00% | 0.00% | 0.00% | 100.00% |
| Javiana | 98.78% | 0.00% | 1.05% | 0.00% | 0.00% | 0.09% | 0.00% | 0.00% | 0.00% | 0.00% | 0.00% | 0.09% | 100.00% |
| Kentucky | 93.79% | 0.05% | 0.05% | 1.25% | 0.09% | 0.32% | 0.00% | 0.00% | 4.45% | 0.00% | 0.00% | 0.00% | 100.00% |
| Mbandaka | 98.51% | 0.30% | 0.15% | 0.90% | 0.00% | 0.00% | 0.00% | 0.15% | 0.00% | 0.00% | 0.00% | 0.00% | 100.00% |
| Montevideo | 97.61% | 0.18% | 0.53% | 0.27% | 0.09% | 0.35% | 0.27% | 0.71% | 0.00% | 0.00% | 0.00% | 0.00% | 100.00% |
| Muenchen | 98.47% | 0.07% | 0.90% | 0.00% | 0.00% | 0.07% | 0.00% | 0.00% | 0.00% | 0.00% | 0.49% | 0.00% | 100.00% |
| Newport | 98.05% | 0.11% | 0.96% | 0.55% | 0.15% | 0.07% | 0.00% | 0.04% | 0.04% | 0.00% | 0.04% | 0.00% | 100.00% |
| Others | 94.36% | 0.33% | 2.73% | 0.58% | 0.39% | 0.49% | 0.55% | 0.11% | 0.06% | 0.08% | 0.27% | 0.04% | 100.00% |
| Reading | 96.58% | 0.00% | 0.54% | 2.52% | 0.18% | 0.18% | 0.00% | 0.00% | 0.00% | 0.00% | 0.00% | 0.00% | 100.00% |
| Saintpaul | 80.24% | 1.86% | 0.87% | 2.18% | 14.30% | 0.44% | 0.00% | 0.11% | 0.00% | 0.00% | 0.00% | 0.00% | 100.00% |
| Schwarzengrund | 92.62% | 0.67% | 0.34% | 1.51% | 2.68% | 2.18% | 0.00% | 0.00% | 0.00% | 0.00% | 0.00% | 0.00% | 100.00% |
| Senftenberg | 89.16% | 0.12% | 2.42% | 1.61% | 0.00% | 0.92% | 2.19% | 1.04% | 0.00% | 0.00% | 2.54% | 0.00% | 100.00% |
| Thompson | 96.28% | 0.00% | 2.38% | 0.00% | 0.00% | 1.19% | 0.00% | 0.15% | 0.00% | 0.00% | 0.00% | 0.00% | 100.00% |
| Typhimurium | 91.93% | 1.54% | 1.31% | 1.61% | 1.58% | 1.23% | 0.00% | 0.32% | 0.05% | 0.00% | 0.02% | 0.42% | 100.00% |
| **Grand Total** | **92.22%** | **2.55%** | **1.61%** | **1.11%** | **0.80%** | **0.57%** | **0.27%** | **0.25%** | **0.23%** | **0.17%** | **0.15%** | **0.07%** | **100.00%** |

**Note**: The percentage (proportion) of ARGs was calculated by the number of positive-predicted ARGs (each cell) divided by the total number of isolates (each row)

## SUPPLEMENTARY DATA S4. THE PROPORTION (%) OF β-LACTAM RESISTANCE GENE PROFILES IN *SALMONELLA ENTERICA* IN THIS STUDY

### The proportion (%) of β-lactam resistance gene profiles in *Salmonella enterica* divided by isolation sources

| Sources/$^{*}$Beta-lactar | $^{1}$None | $^{2}bla_{TEM-1B}$ | $^{3}bla_{CMY-2}$ | $^{4}bla_{CTX-M-65}$ | $^{5}$Others | $^{6}bla_{CARB-2}$ | $^{7}bla_{CMY-2}$, bl | $^{8}bla_{CMY-2}$, bl | $^{9}bla_{TEM-1C}$ | $^{10}bla_{TEM-1A}$ | $^{11}bla_{TEM-116}$ | $^{12}bla_{CTX-M-1}$ | Grand Total |
|---|---|---|---|---|---|---|---|---|---|---|---|---|---|
| Avian | 79.21% | 6.59% | 3.64% | 5.94% | 1.51% | 0.22% | 0.03% | 0.67% | 1.68% | 0.22% | 0.02% | 0.27% | 100.00% |
| Bovine | 72.26% | 3.25% | 11.85% | 0.03% | 1.92% | 2.24% | 6.79% | 1.07% | 0.03% | 0.52% | 0.03% | 0.00% | 100.00% |
| Environmental | 91.83% | 2.85% | 2.32% | 1.08% | 0.50% | 0.44% | 0.27% | 0.18% | 0.30% | 0.14% | 0.09% | 0.00% | 100.00% |
| Feed | 88.30% | 1.89% | 2.07% | 0.86% | 3.96% | 2.07% | 0.00% | 0.00% | 0.00% | 0.69% | 0.17% | 0.00% | 100.00% |
| Food | 93.27% | 2.79% | 1.86% | 0.19% | 0.46% | 0.60% | 0.09% | 0.05% | 0.19% | 0.14% | 0.37% | 0.00% | 100.00% |
| Human | 83.62% | 10.17% | 1.56% | 0.39% | 2.22% | 1.17% | 0.14% | 0.25% | 0.09% | 0.05% | 0.22% | 0.12% | 100.00% |
| Nut/Bean | 98.41% | 0.53% | 0.71% | 0.00% | 0.00% | 0.00% | 0.00% | 0.00% | 0.00% | 0.18% | 0.18% | 0.00% | 100.00% |
| Others | 92.00% | 2.80% | 2.03% | 0.15% | 1.36% | 1.40% | 0.00% | 0.04% | 0.07% | 0.15% | 0.00% | 0.00% | 100.00% |
| Plant | 99.55% | 0.00% | 0.00% | 0.00% | 0.15% | 0.30% | 0.00% | 0.00% | 0.00% | 0.00% | 0.00% | 0.00% | 100.00% |
| Swine | 63.21% | 22.02% | 3.47% | 0.19% | 3.50% | 4.46% | 0.22% | 2.45% | 0.00% | 0.29% | 0.06% | 0.13% | 100.00% |
| Water | 97.85% | 0.16% | 0.36% | 0.63% | 0.70% | 0.16% | 0.00% | 0.00% | 0.02% | 0.09% | 0.02% | 0.00% | 100.00% |
| **Grand Total** | **84.22%** | **6.78%** | **2.82%** | **1.68%** | **1.61%** | **1.03%** | **0.54%** | **0.48%** | **0.46%** | **0.17%** | **0.11%** | **0.10%** | **100.00%** |

| * | β-lactam resistance gene profiles |
|---|---|
| 1 | None; |
| 2 | $bla_{TEM-1B}$; |
| 3 | $bla_{CMY-2}$; |
| 4 | $bla_{CTX-M-65}$; |
| 5 | Others; |
| 6 | $bla_{CARB-2}$; |
| 7 | $bla_{CMY-2}$, $bla_{TEM-1B}$, $bla_{TEM-206}$; |
| 8 | $bla_{CMY-2}$, $bla_{TEM-1B}$; |
| 9 | $bla_{TEM-1C}$; |
| 10 | $bla_{TEM-1A}$; |
| 11 | $bla_{TEM-116}$; |
| 12 | $bla_{CTX-M-1}$; |

### The proportion (%) of β-lactam resistance gene profiles in *Salmonella enterica* divided by serovars

| Serovars/$^{*}$Beta-lacta | $^{1}$None | $^{2}bla_{TEM-1B}$ | $^{3}bla_{CMY-2}$ | $^{4}bla_{CTX-M-65}$ | $^{5}$Others | $^{6}bla_{CARB-2}$ | $^{7}bla_{CMY-2}$, bl | $^{8}bla_{CMY-2}$, bl | $^{9}bla_{TEM-1C}$ | $^{10}bla_{TEM-1A}$ | $^{11}bla_{TEM-116}$ | $^{12}bla_{CTX-M-1}$ | Grand Total |
|---|---|---|---|---|---|---|---|---|---|---|---|---|---|
| Agona | 84.96% | 5.62% | 5.62% | 0.00% | 1.96% | 0.73% | 0.24% | 0.61% | 0.00% | 0.12% | 0.12% | 0.00% | 100.00% |
| Anatum | 90.93% | 2.52% | 0.99% | 0.00% | 3.86% | 0.00% | 0.00% | 0.27% | 0.09% | 1.08% | 0.27% | 0.00% | 100.00% |
| Braenderup | 97.62% | 0.00% | 2.07% | 0.00% | 0.16% | 0.00% | 0.00% | 0.16% | 0.00% | 0.00% | 0.00% | 0.00% | 100.00% |
| Derby | 84.64% | 5.41% | 7.16% | 0.00% | 2.27% | 0.00% | 0.00% | 0.00% | 0.00% | 0.17% | 0.35% | 0.00% | 100.00% |
| Dublin | 27.06% | 2.32% | 26.05% | 0.00% | 4.92% | 0.00% | 35.02% | 2.17% | 0.00% | 2.46% | 0.00% | 0.00% | 100.00% |
| Enteritidis | 92.43% | 5.59% | 0.23% | 0.00% | 1.42% | 0.00% | 0.00% | 0.03% | 0.02% | 0.00% | 0.27% | 0.02% | 100.00% |
| Heidelberg | 71.77% | 10.39% | 13.56% | 0.00% | 1.59% | 0.00% | 0.00% | 2.14% | 0.00% | 0.24% | 0.00% | 0.32% | 100.00% |
| I 1,4,[5],12:i:- | 89.97% | 3.24% | 1.77% | 0.29% | 3.83% | 0.44% | 0.00% | 0.00% | 0.15% | 0.00% | 0.29% | 0.00% | 100.00% |
| Infantis | 61.94% | 4.17% | 1.00% | 30.32% | 0.57% | 0.04% | 0.00% | 0.27% | 0.00% | 0.11% | 0.11% | 1.45% | 100.00% |
| Javiana | 98.95% | 0.61% | 0.09% | 0.00% | 0.17% | 0.00% | 0.00% | 0.00% | 0.00% | 0.09% | 0.00% | 0.09% | 100.00% |
| Kentucky | 90.87% | 3.75% | 4.87% | 0.00% | 0.42% | 0.00% | 0.00% | 0.00% | 0.00% | 0.05% | 0.05% | 0.00% | 100.00% |
| Mbandaka | 99.40% | 0.00% | 0.15% | 0.00% | 0.45% | 0.00% | 0.00% | 0.00% | 0.00% | 0.00% | 0.00% | 0.00% | 100.00% |
| Montevideo | 98.41% | 0.00% | 0.97% | 0.09% | 0.09% | 0.00% | 0.00% | 0.00% | 0.09% | 0.27% | 0.09% | 0.00% | 100.00% |
| Muenchen | 98.96% | 0.14% | 0.28% | 0.00% | 0.21% | 0.00% | 0.00% | 0.07% | 0.00% | 0.14% | 0.21% | 0.00% | 100.00% |
| Newport | 86.87% | 0.92% | 10.52% | 0.00% | 0.48% | 0.92% | 0.00% | 0.07% | 0.00% | 0.15% | 0.07% | 0.00% | 100.00% |
| Others | 93.71% | 3.50% | 0.56% | 0.00% | 1.34% | 0.17% | 0.04% | 0.17% | 0.27% | 0.13% | 0.09% | 0.03% | 100.00% |
| Reading | 61.69% | 0.72% | 1.98% | 0.00% | 3.06% | 0.00% | 0.54% | 0.18% | 31.12% | 0.54% | 0.00% | 0.18% | 100.00% |
| Saintpaul | 63.86% | 28.49% | 1.97% | 0.00% | 3.60% | 0.11% | 0.00% | 1.97% | 0.00% | 0.00% | 0.00% | 0.00% | 100.00% |
| Schwarzengrund | 93.12% | 5.37% | 0.84% | 0.00% | 0.34% | 0.00% | 0.00% | 0.00% | 0.00% | 0.34% | 0.00% | 0.00% | 100.00% |
| Senftenberg | 94.00% | 2.54% | 0.92% | 0.12% | 2.31% | 0.00% | 0.12% | 0.00% | 0.00% | 0.00% | 0.00% | 0.00% | 100.00% |
| Thompson | 97.92% | 0.15% | 0.30% | 0.00% | 1.34% | 0.00% | 0.00% | 0.15% | 0.15% | 0.00% | 0.00% | 0.00% | 100.00% |
| Typhimurium | 54.24% | 27.08% | 5.11% | 0.02% | 3.62% | 7.53% | 0.04% | 2.14% | 0.04% | 0.16% | 0.04% | 0.00% | 100.00% |
| **Grand Total** | **84.22%** | **6.78%** | **2.82%** | **1.68%** | **1.61%** | **1.03%** | **0.54%** | **0.48%** | **0.46%** | **0.17%** | **0.11%** | **0.10%** | **100.00%** |

Note: The percentage (proportion) of ARGs was calculated by the number of positive-predicted ARGs (each cell) divided by the total number of isolates (each row)

SUPPLEMENTARY DATA S5. THE PROPORTION (%) OF FLUOROQUINOLONE RESISTANCE GENE PROFILES IN *SALMONELLA ENTERICA* IN THIS STUDY

**The proportion (%) of fluoroquinolone resistance gene profiles in *Salmonella enterica* divided by isolation sources**

| Sources/[*]Fluoroquin | [1]None | [2]*parC*[T57S | [3]Others | [4]*aac(6')-Ib-* | [5]*qnrB19* | [6]*parC* [T57S | [7]*qnrS1* | [8]*parC*[T57S | [9]*gyrA*[S83F | [10]*parC*[T57S | [11]*oqxA, oqx* | [12]*gyrA*[S83Y | Grand Total |
|---|---|---|---|---|---|---|---|---|---|---|---|---|---|
| Avian | 96.40% | 0.45% | 0.51% | 1.78% | 0.37% | 0.20% | 0.06% | 0.11% | 0.00% | 0.00% | 0.06% | 0.07% | 100.00% |
| Bovine | 97.40% | 1.62% | 0.29% | 0.06% | 0.19% | 0.23% | 0.00% | 0.00% | 0.00% | 0.19% | 0.00% | 0.00% | 100.00% |
| Environmental | 97.98% | 1.03% | 0.25% | 0.18% | 0.18% | 0.39% | 0.00% | 0.00% | 0.00% | 0.00% | 0.00% | 0.00% | 100.00% |
| Feed | 98.28% | 0.69% | 0.17% | 0.52% | 0.00% | 0.00% | 0.00% | 0.00% | 0.00% | 0.34% | 0.00% | 0.00% | 100.00% |
| Food | 96.43% | 1.11% | 0.28% | 1.72% | 0.09% | 0.19% | 0.00% | 0.00% | 0.00% | 0.05% | 0.14% | 0.00% | 100.00% |
| Human | 96.25% | 0.65% | 1.07% | 0.07% | 0.68% | 0.29% | 0.39% | 0.11% | 0.22% | 0.14% | 0.09% | 0.04% | 100.00% |
| Nut/Bean | 99.65% | 0.18% | 0.00% | 0.00% | 0.00% | 0.00% | 0.00% | 0.00% | 0.00% | 0.00% | 0.00% | 0.18% | 100.00% |
| Others | 97.68% | 0.41% | 0.88% | 0.11% | 0.00% | 0.52% | 0.15% | 0.15% | 0.00% | 0.00% | 0.04% | 0.07% | 100.00% |
| Plant | 99.24% | 0.60% | 0.00% | 0.00% | 0.00% | 0.00% | 0.15% | 0.00% | 0.00% | 0.00% | 0.00% | 0.00% | 100.00% |
| Swine | 90.32% | 3.41% | 2.61% | 0.10% | 1.69% | 0.29% | 1.21% | 0.10% | 0.00% | 0.00% | 0.10% | 0.19% | 100.00% |
| Water | 99.12% | 0.38% | 0.47% | 0.00% | 0.00% | 0.02% | 0.00% | 0.00% | 0.00% | 0.00% | 0.00% | 0.00% | 100.00% |
| **Grand Total** | **96.64%** | **0.87%** | **0.76%** | **0.54%** | **0.43%** | **0.25%** | **0.22%** | **0.07%** | **0.07%** | **0.06%** | **0.05%** | **0.05%** | **100.00%** |

| * | Fluoroquinolone resistance gene profiles |
|---|---|
| 1 | None; |
| 2 | *parC*[T57S], *qnrB19*; |
| 3 | Others; |
| 4 | *aac(6')-Ib-cr*, *parC*[T57S]; |
| 5 | *qnrB19*; |
| 6 | *parC*[T57S], *qnrS1*; |
| 7 | *qnrS1*; |
| 8 | *parC*[T57S], *qnrB4*; |
| 9 | *gyrA*[S83F], *qnrS*; |
| 10 | *parC*[T57S], qnrA1; |
| 11 | *oqxA* , *oqxB* ; |
| 12 | *gyrA*[S83Y], *parC*[T57S], *qnrS1*; |

**The proportion (%) of fluoroquinolone resistance gene profiles in *Salmonella enterica* divided by serovars**

| Serovars/[*]Fluoroqui | [1]None | [2]*parC*[T57S | [3]Others | [4]*aac(6')-Ib-* | [5]*qnrB19* | [6]*parC* [T57S | [7]*qnrS1* | [8]*parC*[T57S | [9]*gyrA*[S83F | [10]*parC*[T57S | [11]*oqxA, oqx* | [12]*gyrA*[S83Y | Grand Total |
|---|---|---|---|---|---|---|---|---|---|---|---|---|---|
| Agona | 96.21% | 0.61% | 1.22% | 0.00% | 0.00% | 1.71% | 0.00% | 0.00% | 0.00% | 0.24% | 0.00% | 0.00% | 100.00% |
| Anatum | 95.15% | 1.17% | 0.99% | 0.09% | 0.00% | 0.09% | 0.00% | 2.52% | 0.00% | 0.00% | 0.00% | 0.00% | 100.00% |
| Braenderup | 98.25% | 1.27% | 0.48% | 0.00% | 0.00% | 0.00% | 0.00% | 0.00% | 0.00% | 0.00% | 0.00% | 0.00% | 100.00% |
| Derby | 93.37% | 3.14% | 2.44% | 0.17% | 0.00% | 0.87% | 0.00% | 0.00% | 0.00% | 0.00% | 0.00% | 0.00% | 100.00% |
| Dublin | 99.71% | 0.00% | 0.14% | 0.00% | 0.14% | 0.00% | 0.00% | 0.00% | 0.00% | 0.00% | 0.00% | 0.00% | 100.00% |
| Enteritidis | 99.06% | 0.00% | 0.38% | 0.00% | 0.52% | 0.00% | 0.05% | 0.00% | 0.00% | 0.00% | 0.00% | 0.00% | 100.00% |
| Heidelberg | 83.19% | 6.66% | 0.56% | 9.60% | 0.00% | 0.00% | 0.00% | 0.00% | 0.00% | 0.00% | 0.00% | 0.00% | 100.00% |
| I 1,4,[5],12:i:- | 99.26% | 0.00% | 0.15% | 0.00% | 0.59% | 0.00% | 0.00% | 0.00% | 0.00% | 0.00% | 0.00% | 0.00% | 100.00% |
| Infantis | 99.08% | 0.31% | 0.57% | 0.04% | 0.00% | 0.00% | 0.00% | 0.00% | 0.00% | 0.00% | 0.00% | 0.00% | 100.00% |
| Javiana | 97.99% | 1.84% | 0.09% | 0.00% | 0.00% | 0.09% | 0.00% | 0.00% | 0.00% | 0.00% | 0.00% | 0.00% | 100.00% |
| Kentucky | 98.84% | 0.32% | 0.60% | 0.00% | 0.00% | 0.23% | 0.00% | 0.00% | 0.00% | 0.00% | 0.00% | 0.00% | 100.00% |
| Mbandaka | 99.85% | 0.15% | 0.00% | 0.00% | 0.00% | 0.00% | 0.00% | 0.00% | 0.00% | 0.00% | 0.00% | 0.00% | 100.00% |
| Montevideo | 98.94% | 0.27% | 0.09% | 0.62% | 0.00% | 0.09% | 0.00% | 0.00% | 0.00% | 0.00% | 0.00% | 0.00% | 100.00% |
| Muenchen | 99.24% | 0.76% | 0.00% | 0.00% | 0.00% | 0.00% | 0.00% | 0.00% | 0.00% | 0.00% | 0.00% | 0.00% | 100.00% |
| Newport | 98.05% | 0.52% | 0.22% | 0.00% | 0.00% | 0.33% | 0.00% | 0.00% | 0.00% | 0.77% | 0.00% | 0.11% | 100.00% |
| Others | 95.55% | 1.29% | 0.86% | 0.73% | 0.33% | 0.55% | 0.24% | 0.00% | 0.22% | 0.04% | 0.05% | 0.14% | 100.00% |
| Reading | 99.46% | 0.18% | 0.18% | 0.00% | 0.18% | 0.00% | 0.00% | 0.00% | 0.00% | 0.00% | 0.00% | 0.00% | 100.00% |
| Saintpaul | 98.14% | 0.11% | 0.76% | 0.00% | 0.00% | 0.00% | 0.98% | 0.00% | 0.00% | 0.00% | 0.00% | 0.00% | 100.00% |
| Schwarzengrund | 96.48% | 1.01% | 0.67% | 1.01% | 0.00% | 0.84% | 0.00% | 0.00% | 0.00% | 0.00% | 0.00% | 0.00% | 100.00% |
| Senftenberg | 92.50% | 3.81% | 1.27% | 2.19% | 0.00% | 0.00% | 0.00% | 0.00% | 0.00% | 0.00% | 0.23% | 0.00% | 100.00% |
| Thompson | 98.81% | 0.00% | 0.30% | 0.00% | 0.00% | 0.00% | 0.00% | 0.89% | 0.00% | 0.00% | 0.00% | 0.00% | 100.00% |
| Typhimurium | 94.78% | 0.00% | 1.86% | 0.00% | 2.08% | 0.00% | 1.00% | 0.00% | 0.00% | 0.00% | 0.28% | 0.00% | 100.00% |
| **Grand Total** | **96.64%** | **0.87%** | **0.76%** | **0.54%** | **0.43%** | **0.25%** | **0.22%** | **0.07%** | **0.07%** | **0.06%** | **0.05%** | **0.05%** | **100.00%** |

Note: The percentage (proportion) of ARGs was calculated by the number of positive-predicted ARGs (each cell) divided by the total number of isolates (each row)

**SUPPLEMENTARY DATA S6. THE PROPORTION (%) OF FOLATE PATHWAY ANTAGONIST RESISTANCE GENE PROFILES IN *SALMONELLA ENTERICA* IN THIS STUDY**

**The proportion (%) of folate pathway antagonist resistance gene profiles in *Salmonella enterica* divided by isolation sources**

| Sources/[*]Folate path | [1]None | [2]*sul2* | [3]*sul1* | [4]Others | [5]*dfrA14, su* | [6]*sul1, sul2* | [7]*dfrA7, sul1* | [8]*dfrA1, sul1* | [9]*dfrA14, su* | [10]*dfrA12, su* | [11]dfrA34, su | [12]*dfrA14* | Grand Total |
|---|---|---|---|---|---|---|---|---|---|---|---|---|---|
| Avian | 72.82% | 9.49% | 8.66% | 2.11% | 5.06% | 0.76% | 0.01% | 0.15% | 0.34% | 0.14% | 0.00% | 0.47% | 100.00% |
| Bovine | 67.81% | 22.60% | 3.90% | 1.33% | 0.06% | 1.59% | 0.00% | 0.65% | 0.06% | 0.91% | 1.07% | 0.00% | 100.00% |
| Environmental | 89.53% | 5.10% | 2.14% | 1.33% | 1.06% | 0.28% | 0.02% | 0.07% | 0.18% | 0.04% | 0.23% | 0.02% | 100.00% |
| Feed | 85.89% | 3.79% | 4.65% | 3.96% | 0.34% | 0.34% | 0.00% | 0.69% | 0.00% | 0.17% | 0.00% | 0.17% | 100.00% |
| Food | 90.11% | 3.53% | 3.76% | 1.25% | 0.42% | 0.37% | 0.00% | 0.19% | 0.23% | 0.00% | 0.09% | 0.05% | 100.00% |
| Human | 83.55% | 8.34% | 2.40% | 2.82% | 0.45% | 0.29% | 1.00% | 0.41% | 0.22% | 0.17% | 0.20% | 0.17% | 100.00% |
| Nut/Bean | 98.59% | 0.88% | 0.00% | 0.18% | 0.00% | 0.00% | 0.00% | 0.00% | 0.00% | 0.35% | 0.00% | 0.00% | 100.00% |
| Others | 90.93% | 3.69% | 2.10% | 2.06% | 0.29% | 0.26% | 0.00% | 0.07% | 0.18% | 0.26% | 0.04% | 0.11% | 100.00% |
| Plant | 99.40% | 0.00% | 0.60% | 0.00% | 0.00% | 0.00% | 0.00% | 0.00% | 0.00% | 0.00% | 0.00% | 0.00% | 100.00% |
| Swine | 56.62% | 17.50% | 9.87% | 11.78% | 0.16% | 1.59% | 0.00% | 0.32% | 0.64% | 0.70% | 0.64% | 0.19% | 100.00% |
| Water | 96.20% | 1.33% | 0.75% | 0.45% | 1.17% | 0.05% | 0.00% | 0.00% | 0.00% | 0.00% | 0.00% | 0.05% | 100.00% |
| **Grand Total** | **81.37%** | **8.35%** | **4.23%** | **2.59%** | **1.56%** | **0.54%** | **0.30%** | **0.25%** | **0.23%** | **0.21%** | **0.20%** | **0.18%** | **100.00%** |

| * | Folate pathway antagonist resistance gene profiles |
|---|---|
| 1 | None; |
| 2 | *sul2*; |
| 3 | *sul1*; |
| 4 | Others; |
| 5 | *dfrA14, sul1;* |
| 6 | *sul1, sul2;* |
| 7 | *dfrA7, sul1, sul2;* |
| 8 | *dfrA1, sul1;* |
| 9 | *dfrA14, sul2;* |
| 10 | *dfrA12, sul1, sul2;* |
| 11 | *dfrA34, sul1, sul2;* |
| 12 | *dfrA14;* |

**The proportion (%) of folate pathway antagonist resistance gene profiles in *Salmonella enterica* divided by serovars**

| Serovars/[*]Folate pat | [1]None | [2]*sul2* | [3]*sul1* | [4]Others | [5]*dfrA14, su* | [6]*sul1, sul2* | [7]*dfrA7, sul1* | [8]*dfrA1, sul1* | [9]*dfrA14, su* | [10]*dfrA12, su* | [11]dfrA34, su | [12]*dfrA14* | Grand Total |
|---|---|---|---|---|---|---|---|---|---|---|---|---|---|
| Agona | 75.55% | 7.58% | 6.97% | 4.77% | 0.00% | 0.73% | 0.00% | 0.00% | 1.22% | 0.61% | 1.96% | 0.61% | 100.00% |
| Anatum | 91.28% | 1.71% | 0.45% | 5.93% | 0.00% | 0.09% | 0.00% | 0.00% | 0.00% | 0.54% | 0.00% | 0.00% | 100.00% |
| Braenderup | 97.30% | 0.32% | 1.75% | 0.64% | 0.00% | 0.00% | 0.00% | 0.00% | 0.00% | 0.00% | 0.00% | 0.00% | 100.00% |
| Derby | 59.16% | 4.36% | 30.02% | 4.89% | 0.00% | 0.70% | 0.00% | 0.17% | 0.00% | 0.52% | 0.00% | 0.17% | 100.00% |
| Dublin | 22.72% | 73.23% | 2.17% | 0.14% | 0.00% | 1.45% | 0.00% | 0.14% | 0.00% | 0.14% | 0.00% | 0.00% | 100.00% |
| Enteritidis | 94.49% | 4.23% | 0.14% | 0.75% | 0.00% | 0.11% | 0.00% | 0.23% | 0.06% | 0.00% | 0.00% | 0.00% | 100.00% |
| Heidelberg | 67.57% | 3.33% | 19.35% | 1.90% | 0.00% | 1.43% | 0.16% | 0.00% | 0.24% | 0.32% | 5.71% | 0.00% | 100.00% |
| I 1,4,[5],12:i:- | 91.89% | 2.06% | 2.06% | 3.54% | 0.00% | 0.15% | 0.00% | 0.00% | 0.00% | 0.29% | 0.00% | 0.00% | 100.00% |
| Infantis | 40.39% | 0.31% | 23.74% | 4.25% | 28.33% | 0.46% | 0.00% | 0.42% | 0.00% | 0.00% | 0.00% | 2.11% | 100.00% |
| Javiana | 99.65% | 0.17% | 0.00% | 0.17% | 0.00% | 0.00% | 0.00% | 0.00% | 0.00% | 0.00% | 0.00% | 0.00% | 100.00% |
| Kentucky | 91.94% | 0.97% | 5.33% | 0.88% | 0.00% | 0.00% | 0.00% | 0.05% | 0.83% | 0.00% | 0.00% | 0.00% | 100.00% |
| Mbandaka | 94.33% | 0.15% | 2.54% | 2.84% | 0.00% | 0.15% | 0.00% | 0.00% | 0.00% | 0.00% | 0.00% | 0.00% | 100.00% |
| Montevideo | 97.52% | 1.15% | 0.44% | 0.44% | 0.00% | 0.27% | 0.00% | 0.09% | 0.00% | 0.09% | 0.00% | 0.00% | 100.00% |
| Muenchen | 86.75% | 12.90% | 0.00% | 0.21% | 0.00% | 0.00% | 0.00% | 0.00% | 0.14% | 0.00% | 0.00% | 0.00% | 100.00% |
| Newport | 86.64% | 10.85% | 0.33% | 0.37% | 0.00% | 0.22% | 0.00% | 1.10% | 0.04% | 0.37% | 0.07% | 0.00% | 100.00% |
| Others | 92.46% | 1.65% | 1.03% | 2.89% | 0.00% | 0.17% | 0.99% | 0.22% | 0.26% | 0.12% | 0.03% | 0.17% | 100.00% |
| Reading | 69.42% | 24.82% | 1.80% | 0.72% | 0.00% | 2.34% | 0.00% | 0.00% | 0.90% | 0.00% | 0.00% | 0.00% | 100.00% |
| Saintpaul | 94.00% | 1.09% | 2.29% | 0.76% | 0.00% | 0.66% | 0.00% | 0.00% | 0.87% | 0.33% | 0.00% | 0.00% | 100.00% |
| Schwarzengrund | 87.58% | 8.56% | 1.01% | 1.68% | 0.00% | 0.34% | 0.00% | 0.00% | 0.00% | 0.84% | 0.00% | 0.00% | 100.00% |
| Senftenberg | 92.85% | 1.50% | 2.54% | 1.96% | 0.00% | 0.12% | 0.00% | 0.69% | 0.12% | 0.12% | 0.12% | 0.00% | 100.00% |
| Thompson | 98.07% | 0.15% | 0.15% | 1.64% | 0.00% | 0.00% | 0.00% | 0.00% | 0.00% | 0.00% | 0.00% | 0.00% | 100.00% |
| Typhimurium | 44.80% | 35.77% | 8.96% | 6.53% | 0.00% | 2.45% | 0.00% | 0.35% | 0.35% | 0.74% | 0.02% | 0.04% | 100.00% |
| **Grand Total** | **81.37%** | **8.35%** | **4.23%** | **2.59%** | **1.56%** | **0.54%** | **0.30%** | **0.25%** | **0.23%** | **0.21%** | **0.20%** | **0.18%** | **100.00%** |

Note: The percentage (proportion) of ARGs was calculated by the number of positive-predicted ARGs (each cell) divided by the total number of isolates (each row)

## SUPPLEMENTARY DATA S7. THE PROPORTION (%) OF MACROLIDE RESISTANCE GENE PROFILES IN *SALMONELLA ENTERICA* IN THIS STUDY

**The proportion (%) of macrolide resistance gene profiles in *Salmonella enterica* divided by isolation sources**

| **Sources/[*]Macrolide r** | **[1]None** | **[2]*mph*(A)** | **[3]*mef*(B)** | **[4]*msr*(E)** | **[5]*mph*(A), m** | **[6]*mef*(B), mp** | **[7]*mef*(A), ms** | **[8]*mef*(B), ms** | **Grand Total** |
|---|---|---|---|---|---|---|---|---|---|
| Avian | 99.64% | 0.27% | 0.07% | 0.00% | 0.00% | 0.01% | 0.00% | 0.00% | 100.00% |
| Bovine | 99.42% | 0.49% | 0.06% | 0.00% | 0.00% | 0.00% | 0.03% | 0.00% | 100.00% |
| Environmental | 99.77% | 0.07% | 0.16% | 0.00% | 0.00% | 0.00% | 0.00% | 0.00% | 100.00% |
| Feed | 99.48% | 0.52% | 0.00% | 0.00% | 0.00% | 0.00% | 0.00% | 0.00% | 100.00% |
| Food | 99.77% | 0.23% | 0.00% | 0.00% | 0.00% | 0.00% | 0.00% | 0.00% | 100.00% |
| Human | 99.36% | 0.56% | 0.02% | 0.03% | 0.02% | 0.00% | 0.00% | 0.00% | 100.00% |
| Nut/Bean | 100.00% | 0.00% | 0.00% | 0.00% | 0.00% | 0.00% | 0.00% | 0.00% | 100.00% |
| Others | 99.23% | 0.74% | 0.04% | 0.00% | 0.00% | 0.00% | 0.00% | 0.00% | 100.00% |
| Plant | 100.00% | 0.00% | 0.00% | 0.00% | 0.00% | 0.00% | 0.00% | 0.00% | 100.00% |
| Swine | 98.28% | 0.99% | 0.38% | 0.25% | 0.06% | 0.00% | 0.00% | 0.03% | 100.00% |
| Water | 100.00% | 0.00% | 0.00% | 0.00% | 0.00% | 0.00% | 0.00% | 0.00% | 100.00% |
| **Grand Total** | **99.49%** | **0.39%** | **0.07%** | **0.03%** | **0.01%** | **0.00%** | **0.00%** | **0.00%** | **100.00%** |

| * | **Macrolide resistance gene profiles** |
|---|---|
| 1 | None; |
| 2 | *mph* (A); |
| 3 | *mef* (B); |
| 4 | *msr* (E); |
| 5 | *mph* (A), *msr* (E); |
| 6 | mef(B), *mph* (A); |
| 7 | *mef* (A), *msr* (D); |
| 8 | *mef* (B), *msr* (E); |

**The proportion (%) of macrolide resistance gene profiles in *Salmonella enterica* divided by serovars**

| **Serovars/[*]Macrolide** | **[1]None** | **[2]*mph*(A)** | **[3]*mef*(B)** | **[4]*msr*(E)** | **[5]*mph*(A), m** | **[6]*mef*(B), mp** | **[7]*mef*(A), ms** | **[8]*mef*(B), ms** | **Grand Total** |
|---|---|---|---|---|---|---|---|---|---|
| Agona | 98.04% | 0.86% | 0.86% | 0.12% | 0.12% | 0.00% | 0.00% | 0.00% | 100.00% |
| Anatum | 99.91% | 0.09% | 0.00% | 0.00% | 0.00% | 0.00% | 0.00% | 0.00% | 100.00% |
| Braenderup | 99.84% | 0.16% | 0.00% | 0.00% | 0.00% | 0.00% | 0.00% | 0.00% | 100.00% |
| Derby | 98.95% | 1.05% | 0.00% | 0.00% | 0.00% | 0.00% | 0.00% | 0.00% | 100.00% |
| Dublin | 99.57% | 0.29% | 0.00% | 0.00% | 0.00% | 0.00% | 0.14% | 0.00% | 100.00% |
| Enteritidis | 99.97% | 0.03% | 0.00% | 0.00% | 0.00% | 0.00% | 0.00% | 0.00% | 100.00% |
| Heidelberg | 99.84% | 0.16% | 0.00% | 0.00% | 0.00% | 0.00% | 0.00% | 0.00% | 100.00% |
| I 1,4,[5],12:i:- | 99.71% | 0.29% | 0.00% | 0.00% | 0.00% | 0.00% | 0.00% | 0.00% | 100.00% |
| Infantis | 99.73% | 0.08% | 0.19% | 0.00% | 0.00% | 0.00% | 0.00% | 0.00% | 100.00% |
| Javiana | 99.83% | 0.17% | 0.00% | 0.00% | 0.00% | 0.00% | 0.00% | 0.00% | 100.00% |
| Kentucky | 99.63% | 0.23% | 0.00% | 0.00% | 0.09% | 0.05% | 0.00% | 0.00% | 100.00% |
| Mbandaka | 100.00% | 0.00% | 0.00% | 0.00% | 0.00% | 0.00% | 0.00% | 0.00% | 100.00% |
| Montevideo | 99.91% | 0.09% | 0.00% | 0.00% | 0.00% | 0.00% | 0.00% | 0.00% | 100.00% |
| Muenchen | 99.93% | 0.00% | 0.07% | 0.00% | 0.00% | 0.00% | 0.00% | 0.00% | 100.00% |
| Newport | 98.68% | 1.32% | 0.00% | 0.00% | 0.00% | 0.00% | 0.00% | 0.00% | 100.00% |
| Others | 99.54% | 0.34% | 0.09% | 0.02% | 0.01% | 0.00% | 0.00% | 0.00% | 100.00% |
| Reading | 100.00% | 0.00% | 0.00% | 0.00% | 0.00% | 0.00% | 0.00% | 0.00% | 100.00% |
| Saintpaul | 99.34% | 0.55% | 0.11% | 0.00% | 0.00% | 0.00% | 0.00% | 0.00% | 100.00% |
| Schwarzengrund | 99.50% | 0.50% | 0.00% | 0.00% | 0.00% | 0.00% | 0.00% | 0.00% | 100.00% |
| Senftenberg | 99.77% | 0.23% | 0.00% | 0.00% | 0.00% | 0.00% | 0.00% | 0.00% | 100.00% |
| Thompson | 99.70% | 0.30% | 0.00% | 0.00% | 0.00% | 0.00% | 0.00% | 0.00% | 100.00% |
| Typhimurium | 98.69% | 1.00% | 0.14% | 0.14% | 0.02% | 0.00% | 0.00% | 0.02% | 100.00% |
| **Grand Total** | **99.49%** | **0.39%** | **0.07%** | **0.03%** | **0.01%** | **0.00%** | **0.00%** | **0.00%** | **100.00%** |

Note: The percentage (proportion) of ARGs was calculated by the number of positive-predicted ARGs (each cell) divided by the total number of isolates (each row)

**SUPPLEMENTARY DATA S8. THE PROPORTION (%) OF PHENICOL RESISTANCE GENE PROFILES IN *SALMONELLA ENTERICA* IN THIS STUDY**

**The proportion (%) of phenicol resistance gene profiles in *Salmonella enterica* divided by isolation sources**

| **Sources/[*]Phenicol re** | **[1]None** | **[2]*floR*** | **[3]*catA1*** | **[4]*cmlA1*** | **[5]*cmlA1, flo*** | **[6]Others** | **[7]*catA2*** | **[8]*oqxA, oqx*** | **[9]*cml*** | **[10]*floR, oqx*** | **[11]*cmlA1, fl*** | **[12]cat** | **Grand Total** |
|---|---|---|---|---|---|---|---|---|---|---|---|---|---|
| Avian | 91.45% | 7.25% | 0.13% | 0.42% | 0.25% | 0.23% | 0.07% | 0.10% | 0.00% | 0.03% | 0.04% | 0.03% | 100.00% |
| Bovine | 74.41% | 23.55% | 0.45% | 0.32% | 0.88% | 0.10% | 0.06% | 0.03% | 0.00% | 0.00% | 0.00% | 0.19% | 100.00% |
| Environmental | 94.99% | 4.20% | 0.07% | 0.09% | 0.30% | 0.09% | 0.02% | 0.04% | 0.19% | 0.00% | 0.00% | 0.02% | 100.00% |
| Feed | 94.66% | 5.34% | 0.00% | 0.00% | 0.00% | 0.00% | 0.00% | 0.00% | 0.00% | 0.00% | 0.00% | 0.00% | 100.00% |
| Food | 96.84% | 2.00% | 0.37% | 0.28% | 0.09% | 0.14% | 0.09% | 0.14% | 0.05% | 0.00% | 0.00% | 0.00% | 100.00% |
| Human | 93.81% | 3.55% | 1.34% | 0.28% | 0.22% | 0.49% | 0.08% | 0.04% | 0.00% | 0.13% | 0.07% | 0.01% | 100.00% |
| Nut/Bean | 99.12% | 0.71% | 0.00% | 0.00% | 0.18% | 0.00% | 0.00% | 0.00% | 0.00% | 0.00% | 0.00% | 0.00% | 100.00% |
| Others | 93.81% | 4.79% | 0.11% | 0.29% | 0.55% | 0.11% | 0.22% | 0.04% | 0.04% | 0.00% | 0.00% | 0.04% | 100.00% |
| Plant | 100.00% | 0.00% | 0.00% | 0.00% | 0.00% | 0.00% | 0.00% | 0.00% | 0.00% | 0.00% | 0.00% | 0.00% | 100.00% |
| Swine | 81.57% | 10.47% | 0.38% | 3.25% | 1.43% | 0.92% | 0.80% | 0.16% | 0.76% | 0.06% | 0.16% | 0.03% | 100.00% |
| Water | 98.28% | 1.29% | 0.00% | 0.00% | 0.00% | 0.14% | 0.02% | 0.20% | 0.00% | 0.00% | 0.00% | 0.07% | 100.00% |
| **Grand Total** | **92.06%** | **5.94%** | **0.51%** | **0.45%** | **0.35%** | **0.30%** | **0.12%** | **0.08%** | **0.08%** | **0.05%** | **0.04%** | **0.03%** | **100.00%** |

| * | **Phenicol resistance gene profiles** |
|---|---|
| 1 | None; |
| 2 | *floR* ; |
| 3 | *catA1*; |
| 4 | *cmlA1*; |
| 5 | *cmlA1, floR*; |
| 6 | Others; |
| 7 | *catA2*; |
| 8 | *oqxA* , *oqxB* ; |
| 9 | *cml* ; |
| 10 | *floR* , *oqxA* , *oqxB* ; |
| 11 | *cmlA1, floR, oqxA, oqxB*; |
| 12 | *cat* ; |

**The proportion (%) of phenicol resistance gene profiles in *Salmonella enterica* divided by serovars**

| **Serovars/[*]Phenicol r** | **[1]None** | **[2]*floR*** | **[3]*catA1*** | **[4]*cmlA1*** | **[5]*cmlA1, flo*** | **[6]Others** | **[7]*catA2*** | **[8]*oqxA, oqx*** | **[9]*cml*** | **[10]*floR, oqx*** | **[11]*cmlA1, fl*** | **[12]cat** | **Grand Total** |
|---|---|---|---|---|---|---|---|---|---|---|---|---|---|
| Agona | 90.46% | 7.58% | 0.00% | 0.24% | 0.49% | 0.37% | 0.00% | 0.00% | 0.86% | 0.00% | 0.00% | 0.00% | 100.00% |
| Anatum | 92.72% | 4.40% | 0.09% | 0.18% | 0.54% | 0.99% | 0.18% | 0.27% | 0.36% | 0.00% | 0.00% | 0.27% | 100.00% |
| Braenderup | 99.36% | 0.16% | 0.00% | 0.16% | 0.16% | 0.16% | 0.00% | 0.00% | 0.00% | 0.00% | 0.00% | 0.00% | 100.00% |
| Derby | 93.37% | 3.32% | 0.17% | 0.35% | 0.35% | 0.17% | 0.17% | 1.92% | 0.00% | 0.17% | 0.00% | 0.00% | 100.00% |
| Dublin | 28.22% | 65.12% | 2.46% | 1.01% | 3.18% | 0.00% | 0.00% | 0.00% | 0.00% | 0.00% | 0.00% | 0.00% | 100.00% |
| Enteritidis | 99.39% | 0.27% | 0.03% | 0.05% | 0.03% | 0.12% | 0.02% | 0.05% | 0.00% | 0.02% | 0.02% | 0.02% | 100.00% |
| Heidelberg | 89.77% | 7.53% | 0.40% | 0.71% | 1.27% | 0.08% | 0.24% | 0.00% | 0.00% | 0.00% | 0.00% | 0.00% | 100.00% |
| I 1,4,[5],12:i:- | 98.53% | 1.18% | 0.00% | 0.29% | 0.00% | 0.00% | 0.00% | 0.00% | 0.00% | 0.00% | 0.00% | 0.00% | 100.00% |
| Infantis | 63.40% | 35.64% | 0.04% | 0.77% | 0.00% | 0.15% | 0.00% | 0.00% | 0.00% | 0.00% | 0.00% | 0.00% | 100.00% |
| Javiana | 99.83% | 0.00% | 0.00% | 0.09% | 0.00% | 0.00% | 0.09% | 0.00% | 0.00% | 0.00% | 0.00% | 0.00% | 100.00% |
| Kentucky | 99.49% | 0.19% | 0.05% | 0.09% | 0.00% | 0.09% | 0.00% | 0.05% | 0.00% | 0.00% | 0.00% | 0.05% | 100.00% |
| Mbandaka | 99.55% | 0.30% | 0.15% | 0.00% | 0.00% | 0.00% | 0.00% | 0.00% | 0.00% | 0.00% | 0.00% | 0.00% | 100.00% |
| Montevideo | 98.41% | 0.62% | 0.00% | 0.00% | 0.80% | 0.09% | 0.00% | 0.00% | 0.00% | 0.00% | 0.00% | 0.09% | 100.00% |
| Muenchen | 99.45% | 0.14% | 0.42% | 0.00% | 0.00% | 0.00% | 0.00% | 0.00% | 0.00% | 0.00% | 0.00% | 0.00% | 100.00% |
| Newport | 87.53% | 12.14% | 0.22% | 0.00% | 0.04% | 0.04% | 0.00% | 0.00% | 0.00% | 0.00% | 0.00% | 0.04% | 100.00% |
| Others | 95.96% | 1.18% | 1.29% | 0.44% | 0.32% | 0.32% | 0.11% | 0.10% | 0.19% | 0.04% | 0.00% | 0.05% | 100.00% |
| Reading | 96.94% | 2.70% | 0.00% | 0.00% | 0.18% | 0.00% | 0.18% | 0.00% | 0.00% | 0.00% | 0.00% | 0.00% | 100.00% |
| Saintpaul | 96.83% | 2.51% | 0.11% | 0.44% | 0.00% | 0.11% | 0.00% | 0.00% | 0.00% | 0.00% | 0.00% | 0.00% | 100.00% |
| Schwarzengrund | 98.66% | 0.17% | 0.00% | 0.00% | 1.01% | 0.00% | 0.17% | 0.00% | 0.00% | 0.00% | 0.00% | 0.00% | 100.00% |
| Senftenberg | 97.35% | 0.81% | 0.00% | 0.23% | 0.12% | 0.69% | 0.35% | 0.23% | 0.00% | 0.00% | 0.00% | 0.23% | 100.00% |
| Thompson | 98.51% | 0.15% | 0.00% | 0.45% | 0.15% | 0.15% | 0.60% | 0.00% | 0.00% | 0.00% | 0.00% | 0.00% | 100.00% |
| Typhimurium | 84.19% | 11.03% | 0.32% | 1.63% | 0.84% | 0.98% | 0.39% | 0.07% | 0.00% | 0.26% | 0.30% | 0.00% | 100.00% |
| **Grand Total** | **92.06%** | **5.94%** | **0.51%** | **0.45%** | **0.35%** | **0.30%** | **0.12%** | **0.08%** | **0.08%** | **0.05%** | **0.04%** | **0.03%** | **100.00%** |

Note: The percentage (proportion) of ARGs was calculated by the number of positive-predicted ARGs (each cell) divided by the total number of isolates (each row)

**SUPPLEMENTARY DATA S9. THE PROPORTION (%) OF POLYMYXIN RESISTANCE GENE PROFILES IN *SALMONELLA ENTERICA* IN THIS STUDY**

**The proportion (%) of polymyxin resistance gene profiles in *Salmonella enterica* divided by isolation source**

| Sources/[*]Polymyxin | [1]None | [2]*mcr-9* | [3]*mcr-1.1* | [4]*mcr-5.1* | [5]*mcr-3.1* | [6]*mcr-1.1, m* | [7]*mcr-1.1, m* | [8]*mcr-1.26* | [9]*mcr-4.6* | [10]*mcr-3.20* | [11]*mcr-3.21* | [12]*mcr-1.2* | [13]*mcr-4.2* | Grand Total |
|---|---|---|---|---|---|---|---|---|---|---|---|---|---|---|
| Avian | 96.86% | 2.86% | 0.17% | 0.10% | 0.00% | 0.00% | 0.00% | 0.00% | 0.00% | 0.00% | 0.00% | 0.01% | 0.00% | 100.00% |
| Bovine | 99.55% | 0.39% | 0.00% | 0.03% | 0.00% | 0.00% | 0.00% | 0.00% | 0.00% | 0.00% | 0.00% | 0.00% | 0.03% | 100.00% |
| Environmental | 99.59% | 0.34% | 0.00% | 0.07% | 0.00% | 0.00% | 0.00% | 0.00% | 0.00% | 0.00% | 0.00% | 0.00% | 0.00% | 100.00% |
| Feed | 99.14% | 0.86% | 0.00% | 0.00% | 0.00% | 0.00% | 0.00% | 0.00% | 0.00% | 0.00% | 0.00% | 0.00% | 0.00% | 100.00% |
| Food | 98.19% | 1.76% | 0.05% | 0.00% | 0.00% | 0.00% | 0.00% | 0.00% | 0.00% | 0.00% | 0.00% | 0.00% | 0.00% | 100.00% |
| Human | 99.65% | 0.23% | 0.07% | 0.00% | 0.00% | 0.04% | 0.01% | 0.01% | 0.00% | 0.00% | 0.00% | 0.00% | 0.00% | 100.00% |
| Nut/Bean | 99.82% | 0.18% | 0.00% | 0.00% | 0.00% | 0.00% | 0.00% | 0.00% | 0.00% | 0.00% | 0.00% | 0.00% | 0.00% | 100.00% |
| Others | 99.45% | 0.29% | 0.26% | 0.00% | 0.00% | 0.00% | 0.00% | 0.00% | 0.00% | 0.00% | 0.00% | 0.00% | 0.00% | 100.00% |
| Plant | 100.00% | 0.00% | 0.00% | 0.00% | 0.00% | 0.00% | 0.00% | 0.00% | 0.00% | 0.00% | 0.00% | 0.00% | 0.00% | 100.00% |
| Swine | 97.55% | 1.56% | 0.29% | 0.06% | 0.29% | 0.00% | 0.10% | 0.00% | 0.06% | 0.06% | 0.03% | 0.00% | 0.00% | 100.00% |
| Water | 99.98% | 0.02% | 0.00% | 0.00% | 0.00% | 0.00% | 0.00% | 0.00% | 0.00% | 0.00% | 0.00% | 0.00% | 0.00% | 100.00% |
| **Grand Total** | **98.82%** | **0.99%** | **0.09%** | **0.04%** | **0.02%** | **0.01%** | **0.01%** | **0.00%** | **0.00%** | **0.00%** | **0.00%** | **0.00%** | **0.00%** | **100.00%** |

| * | Polymyxin resistance gene profiles |
|---|---|
| 1 | None; |
| 2 | *mcr-9*; |
| 3 | *mcr-1.1*; |
| 4 | *mcr-5.1*; |
| 5 | *mcr-3.1*; |
| 6 | *mcr-1.1*, *mcr-1.2*, *mcr-1.3*, *mcr-1.8*, *mcr-1.11*, *mcr-1.12*, *mcr-1.14*, *mcr-1.26*, *mcr-1.27*; |
| 7 | *mcr-1.1*, *mcr-9*; |
| 8 | *mcr-1.26*; |
| 9 | *mcr-4.6*; |
| 10 | *mcr-3.20*; |
| 11 | *mcr-3.21*; |
| 12 | *mcr-1.2*; |
| 13 | *mcr-4.2*; |

**The proportion (%) of polymyxin resistance gene profiles in *Salmonella enterica* divided by serovars**

| Serovars/[*]Polymyxir | [1]None | [2]*mcr-9* | [3]*mcr-1.1* | [4]*mcr-5.1* | [5]*mcr-3.1* | [6]*mcr-1.1, m* | [7]*mcr-1.1, m* | [8]*mcr-1.26* | [9]*mcr-4.6* | [10]*mcr-3.20* | [11]*mcr-3.21* | [12]*mcr-1.2* | [13]*mcr-4.2* | Grand Total |
|---|---|---|---|---|---|---|---|---|---|---|---|---|---|---|
| Agona | 98.66% | 0.86% | 0.49% | 0.00% | 0.00% | 0.00% | 0.00% | 0.00% | 0.00% | 0.00% | 0.00% | 0.00% | 0.00% | 100.00% |
| Anatum | 99.01% | 0.45% | 0.36% | 0.00% | 0.00% | 0.00% | 0.18% | 0.00% | 0.00% | 0.00% | 0.00% | 0.00% | 0.00% | 100.00% |
| Braenderup | 99.68% | 0.32% | 0.00% | 0.00% | 0.00% | 0.00% | 0.00% | 0.00% | 0.00% | 0.00% | 0.00% | 0.00% | 0.00% | 100.00% |
| Derby | 100.00% | 0.00% | 0.00% | 0.00% | 0.00% | 0.00% | 0.00% | 0.00% | 0.00% | 0.00% | 0.00% | 0.00% | 0.00% | 100.00% |
| Dublin | 100.00% | 0.00% | 0.00% | 0.00% | 0.00% | 0.00% | 0.00% | 0.00% | 0.00% | 0.00% | 0.00% | 0.00% | 0.00% | 100.00% |
| Enteritidis | 99.97% | 0.00% | 0.02% | 0.00% | 0.00% | 0.00% | 0.00% | 0.02% | 0.00% | 0.00% | 0.00% | 0.00% | 0.00% | 100.00% |
| Heidelberg | 89.93% | 10.07% | 0.00% | 0.00% | 0.00% | 0.00% | 0.00% | 0.00% | 0.00% | 0.00% | 0.00% | 0.00% | 0.00% | 100.00% |
| I 1,4,[5],12:i:- | 99.85% | 0.00% | 0.00% | 0.15% | 0.00% | 0.00% | 0.00% | 0.00% | 0.00% | 0.00% | 0.00% | 0.00% | 0.00% | 100.00% |
| Infantis | 99.92% | 0.04% | 0.04% | 0.00% | 0.00% | 0.00% | 0.00% | 0.00% | 0.00% | 0.00% | 0.00% | 0.00% | 0.00% | 100.00% |
| Javiana | 99.91% | 0.09% | 0.00% | 0.00% | 0.00% | 0.00% | 0.00% | 0.00% | 0.00% | 0.00% | 0.00% | 0.00% | 0.00% | 100.00% |
| Kentucky | 99.95% | 0.05% | 0.00% | 0.00% | 0.00% | 0.00% | 0.00% | 0.00% | 0.00% | 0.00% | 0.00% | 0.00% | 0.00% | 100.00% |
| Mbandaka | 98.96% | 1.04% | 0.00% | 0.00% | 0.00% | 0.00% | 0.00% | 0.00% | 0.00% | 0.00% | 0.00% | 0.00% | 0.00% | 100.00% |
| Montevideo | 99.91% | 0.09% | 0.00% | 0.00% | 0.00% | 0.00% | 0.00% | 0.00% | 0.00% | 0.00% | 0.00% | 0.00% | 0.00% | 100.00% |
| Muenchen | 99.93% | 0.07% | 0.00% | 0.00% | 0.00% | 0.00% | 0.00% | 0.00% | 0.00% | 0.00% | 0.00% | 0.00% | 0.00% | 100.00% |
| Newport | 99.93% | 0.00% | 0.07% | 0.00% | 0.00% | 0.00% | 0.00% | 0.00% | 0.00% | 0.00% | 0.00% | 0.00% | 0.00% | 100.00% |
| Others | 98.71% | 0.96% | 0.14% | 0.11% | 0.01% | 0.04% | 0.00% | 0.00% | 0.01% | 0.00% | 0.00% | 0.01% | 0.00% | 100.00% |
| Reading | 99.82% | 0.18% | 0.00% | 0.00% | 0.00% | 0.00% | 0.00% | 0.00% | 0.00% | 0.00% | 0.00% | 0.00% | 0.00% | 100.00% |
| Saintpaul | 89.41% | 10.59% | 0.00% | 0.00% | 0.00% | 0.00% | 0.00% | 0.00% | 0.00% | 0.00% | 0.00% | 0.00% | 0.00% | 100.00% |
| Schwarzengrund | 98.32% | 1.68% | 0.00% | 0.00% | 0.00% | 0.00% | 0.00% | 0.00% | 0.00% | 0.00% | 0.00% | 0.00% | 0.00% | 100.00% |
| Senftenberg | 98.27% | 1.73% | 0.00% | 0.00% | 0.00% | 0.00% | 0.00% | 0.00% | 0.00% | 0.00% | 0.00% | 0.00% | 0.00% | 100.00% |
| Thompson | 99.26% | 0.74% | 0.00% | 0.00% | 0.00% | 0.00% | 0.00% | 0.00% | 0.00% | 0.00% | 0.00% | 0.00% | 0.00% | 100.00% |
| Typhimurium | 98.55% | 0.96% | 0.21% | 0.04% | 0.12% | 0.00% | 0.04% | 0.02% | 0.00% | 0.04% | 0.02% | 0.00% | 0.02% | 100.00% |
| **Grand Total** | **98.82%** | **0.99%** | **0.09%** | **0.04%** | **0.02%** | **0.01%** | **0.01%** | **0.00%** | **0.00%** | **0.00%** | **0.00%** | **0.00%** | **0.00%** | **100.00%** |

Note: The percentage (proportion) of ARGs was calculated by the number of positive-predicted ARGs (each cell) divided by the total number of isolates (each row)

**SUPPLEMENTARY TABLE S10. THE PROPORTION (%) OF TETRACYCLINE RESISTANCE GENE PROFILES IN *SALMONELLA ENTERICA* IN THIS STUDY**

**The proportion (%) of tetracycline resistance gene profiles in *Salmonella enterica* divided by isolation sources**

| Sources/*Tetracyclin | [1]None | [2]*tet*(A) | [3]*tet*(B) | [4]*tet*(G) | [5]*tet*(C) | [6]Others | [7]*tet*(A), *tet*( | [8]*tet*(A), *tet*( | [9]*tet*(A), *tet*( | [10]*tet*(A), *tet* | [11]*tet*(D) | [12]*tet*(B), *tet* | Grand Total |
|---|---|---|---|---|---|---|---|---|---|---|---|---|---|
| Avian | 56.12% | 26.10% | 15.90% | 0.21% | 0.61% | 0.11% | 0.12% | 0.34% | 0.00% | 0.39% | 0.08% | 0.01% | 100.00% |
| Bovine | 63.36% | 25.56% | 3.93% | 2.44% | 2.18% | 0.68% | 0.42% | 0.36% | 0.75% | 0.10% | 0.10% | 0.13% | 100.00% |
| Environmental | 86.84% | 7.96% | 3.67% | 0.37% | 0.44% | 0.16% | 0.23% | 0.12% | 0.18% | 0.02% | 0.02% | 0.00% | 100.00% |
| Feed | 82.79% | 12.22% | 2.41% | 1.55% | 0.86% | 0.17% | 0.00% | 0.00% | 0.00% | 0.00% | 0.00% | 0.00% | 100.00% |
| Food | 86.86% | 9.19% | 2.97% | 0.37% | 0.23% | 0.23% | 0.05% | 0.00% | 0.09% | 0.00% | 0.00% | 0.00% | 100.00% |
| Human | 84.31% | 7.36% | 6.21% | 0.93% | 0.12% | 0.27% | 0.27% | 0.15% | 0.17% | 0.01% | 0.09% | 0.11% | 100.00% |
| Nut/Bean | 98.06% | 1.77% | 0.00% | 0.00% | 0.00% | 0.00% | 0.18% | 0.00% | 0.00% | 0.00% | 0.00% | 0.00% | 100.00% |
| Others | 89.01% | 5.64% | 2.54% | 1.25% | 0.52% | 0.29% | 0.41% | 0.07% | 0.04% | 0.04% | 0.18% | 0.00% | 100.00% |
| Plant | 98.19% | 0.15% | 1.21% | 0.00% | 0.45% | 0.00% | 0.00% | 0.00% | 0.00% | 0.00% | 0.00% | 0.00% | 100.00% |
| Swine | 46.44% | 16.74% | 23.04% | 3.66% | 4.30% | 2.29% | 1.59% | 1.15% | 0.00% | 0.00% | 0.38% | 0.41% | 100.00% |
| Water | 95.32% | 3.34% | 0.72% | 0.16% | 0.34% | 0.11% | 0.00% | 0.00% | 0.00% | 0.00% | 0.00% | 0.00% | 100.00% |
| **Grand Total** | **76.15%** | **12.95%** | **8.00%** | **0.88%** | **0.74%** | **0.36%** | **0.29%** | **0.24%** | **0.13%** | **0.10%** | **0.09%** | **0.07%** | **100.00%** |

| * | Tetracycline resistance gene profiles |
|---|---|
| 1 | None; |
| 2 | *tet* (A); |
| 3 | *tet* (B); |
| 4 | *tet* (G); |
| 5 | *tet* (C); |
| 6 | Others; |
| 7 | *tet* (A), *tet* (M); |
| 8 | *tet* (A), *tet(* B); |
| 9 | *tet* (A), *tet* (B), *tet(* O); |
| 10 | *tet* (A), *tet* (C); |
| 11 | *tet* (D); |
| 12 | *tet* (B), *tet* (M); |

**The proportion (%) of tetracycline resistance gene profiles in *Salmonella enterica* divided by serovars**

| Serovars/*Tetracycli | [1]None | [2]*tet*(A) | [3]*tet*(B) | [4]*tet*(G) | [5]*tet*(C) | [6]Others | [7]*tet*(A), *tet*( | [8]*tet*(A), *tet*( | [9]*tet*(A), *tet*( | [10]*tet*(A), *tet* | [11]*tet*(D) | [12]*tet*(B), *tet* | Grand Total |
|---|---|---|---|---|---|---|---|---|---|---|---|---|---|
| Agona | 72.98% | 16.87% | 7.21% | 0.37% | 0.24% | 0.12% | 1.22% | 0.49% | 0.00% | 0.00% | 0.49% | 0.00% | 100.00% |
| Anatum | 73.94% | 7.01% | 4.76% | 0.00% | 12.76% | 0.90% | 0.18% | 0.09% | 0.00% | 0.09% | 0.27% | 0.00% | 100.00% |
| Braenderup | 96.82% | 2.54% | 0.16% | 0.16% | 0.16% | 0.00% | 0.00% | 0.00% | 0.00% | 0.00% | 0.16% | 0.00% | 100.00% |
| Derby | 30.19% | 50.96% | 16.93% | 0.17% | 0.52% | 0.17% | 0.52% | 0.35% | 0.00% | 0.00% | 0.17% | 0.00% | 100.00% |
| Dublin | 23.30% | 74.53% | 2.03% | 0.00% | 0.00% | 0.14% | 0.00% | 0.00% | 0.00% | 0.00% | 0.00% | 0.00% | 100.00% |
| Enteritidis | 95.66% | 3.91% | 0.38% | 0.00% | 0.00% | 0.03% | 0.02% | 0.00% | 0.00% | 0.00% | 0.00% | 0.00% | 100.00% |
| Heidelberg | 73.28% | 6.11% | 8.64% | 0.00% | 3.49% | 1.03% | 0.16% | 2.22% | 4.76% | 0.32% | 0.00% | 0.00% | 100.00% |
| I 1,4,[5],12:i:- | 89.53% | 4.87% | 4.87% | 0.44% | 0.00% | 0.15% | 0.00% | 0.00% | 0.00% | 0.00% | 0.15% | 0.00% | 100.00% |
| Infantis | 42.00% | 57.24% | 0.50% | 0.04% | 0.08% | 0.04% | 0.00% | 0.00% | 0.00% | 0.00% | 0.11% | 0.00% | 100.00% |
| Javiana | 99.65% | 0.26% | 0.09% | 0.00% | 0.00% | 0.00% | 0.00% | 0.00% | 0.00% | 0.00% | 0.00% | 0.00% | 100.00% |
| Kentucky | 30.86% | 7.78% | 60.52% | 0.00% | 0.46% | 0.09% | 0.05% | 0.19% | 0.00% | 0.05% | 0.00% | 0.00% | 100.00% |
| Mbandaka | 89.70% | 4.48% | 5.67% | 0.00% | 0.15% | 0.00% | 0.00% | 0.00% | 0.00% | 0.00% | 0.00% | 0.00% | 100.00% |
| Montevideo | 94.43% | 1.50% | 1.59% | 0.00% | 1.41% | 0.18% | 0.80% | 0.00% | 0.00% | 0.09% | 0.00% | 0.00% | 100.00% |
| Muenchen | 86.48% | 13.38% | 0.00% | 0.00% | 0.14% | 0.00% | 0.00% | 0.00% | 0.00% | 0.00% | 0.00% | 0.00% | 100.00% |
| Newport | 85.95% | 13.36% | 0.40% | 0.04% | 0.07% | 0.07% | 0.00% | 0.11% | 0.00% | 0.00% | 0.00% | 0.00% | 100.00% |
| Others | 88.51% | 7.35% | 2.60% | 0.16% | 0.58% | 0.16% | 0.39% | 0.11% | 0.00% | 0.01% | 0.11% | 0.01% | 100.00% |
| Reading | 67.45% | 26.98% | 0.18% | 0.00% | 4.32% | 0.36% | 0.18% | 0.18% | 0.00% | 0.36% | 0.00% | 0.00% | 100.00% |
| Saintpaul | 65.61% | 18.23% | 14.63% | 0.00% | 0.11% | 0.00% | 0.87% | 0.55% | 0.00% | 0.00% | 0.00% | 0.00% | 100.00% |
| Schwarzengrund | 81.71% | 12.42% | 4.53% | 0.00% | 1.01% | 0.00% | 0.00% | 0.17% | 0.00% | 0.00% | 0.17% | 0.00% | 100.00% |
| Senftenberg | 93.54% | 4.73% | 0.69% | 0.00% | 0.00% | 0.69% | 0.12% | 0.00% | 0.00% | 0.00% | 0.23% | 0.00% | 100.00% |
| Thompson | 98.07% | 0.89% | 0.00% | 0.00% | 0.00% | 0.00% | 0.00% | 0.00% | 0.00% | 0.00% | 1.04% | 0.00% | 100.00% |
| Typhimurium | 44.54% | 17.66% | 26.03% | 6.78% | 0.23% | 1.82% | 0.82% | 0.84% | 0.00% | 0.65% | 0.07% | 0.56% | 100.00% |
| **Grand Total** | **76.15%** | **12.95%** | **8.00%** | **0.88%** | **0.74%** | **0.36%** | **0.29%** | **0.24%** | **0.13%** | **0.10%** | **0.09%** | **0.07%** | **100.00%** |

Note: The percentage (proportion) of ARGs was calculated by the number of positive-predicted ARGs (each cell) divided by the total number of isolates (each row)
